# DNA methylation variations underlie lettuce domestication and divergence

**DOI:** 10.1186/s13059-024-03310-x

**Published:** 2024-06-17

**Authors:** Shuai Cao, Nunchanoke Sawettalake, Ping Li, Sheng Fan, Lisha Shen

**Affiliations:** 1 Temasek Life Sciences Laboratory, 1 Research Link, National University of Singapore, Singapore, 117604 Singapore; 2 Department of Biological Sciences, Faculty of Science, National University of Singapore, Singapore, 117543 Singapore

**Keywords:** Lettuce, DNA methylation, Domestication, Divergence, Epigenetic variation

## Abstract

**Background:**

Lettuce (*Lactuca sativa L.*) is an economically important vegetable crop worldwide. Lettuce is believed to be domesticated from a single wild ancestor *Lactuca serriola* and subsequently diverged into two major morphologically distinct vegetable types: leafy lettuce and stem lettuce. However, the role of epigenetic variation in lettuce domestication and divergence remains largely unknown.

**Results:**

To understand the genetic and epigenetic basis underlying lettuce domestication and divergence, we generate single-base resolution DNA methylomes from 52 *Lactuca* accessions, including major lettuce cultivars and wild relatives. We find a significant increase of DNA methylation during lettuce domestication and uncover abundant epigenetic variations associated with lettuce domestication and divergence. Interestingly, DNA methylation variations specifically associated with leafy and stem lettuce are related to regulation and metabolic processes, respectively, while those associated with both types are enriched in stress responses. Moreover, we reveal that domestication-induced DNA methylation changes could influence expression levels of nearby and distal genes possibly through affecting chromatin accessibility and chromatin loop.

**Conclusion:**

Our study provides population epigenomic insights into crop domestication and divergence and valuable resources for further domestication for diversity and epigenetic breeding to boost crop improvement.

**Supplementary Information:**

The online version contains supplementary material available at 10.1186/s13059-024-03310-x.

## Background

Lettuce (*Lactuca sativa* L.), an important representative of the *Asteraceae* family, is one of the most commonly cultivated and consumed vegetable crops worldwide and serves as a prominent natural source of phytonutrients for humans [1]. As a morphologically and genetically diverse vegetable crop, modern lettuce cultivars could be classified into several distinct horticultural types based on their morphological features, including butterhead, crisp, cos (also known as romaine), looseleaf, Latin, stem (also called stalk), and oilseed lettuce [2]. Among these, the former five types are collectively known as the leafy type as their leaves are commonly consumed, while the latter two are referred to as the non-leafy type as stem or seeds are harvested for consumption or oil production in stem or oilseed lettuce, respectively. Leafy and stem lettuce, the two main types of cultivated lettuce, are globally favored vegetables, particularly in the United States, where they rank among the top vegetable crops in terms of production and consumption (https://www.fao.org/). Despite great morphological variations, different horticultural types of lettuce share some common domestication traits, such as absence of leaf prickles, non-shattering seed pods, and late bolting [3, 4]. Different types of cultivated lettuce are believed to originate from a single domestication event from the wild progenitor prickly lettuce (*L. serriola*) near the Caucasus in middle east of Asia at approximately 4000 BC [5–7]. The initial domestication and subsequent diversification of lettuce give rise to distinct plant morphologies, such as leaf and stem shapes and head formation, which also involves genomic introgressions from its wild relative species, like *L. serriola* and *L. virosa* [6, 8, 9]. Three wild relatives of lettuce, *L. serriola*, *L. virosa*, and *L. saligna*, are compatible with cultivated lettuce to different degrees, serving as important sources of novel traits in lettuce breeding [2, 9, 10]. Although abundant genetic variations during lettuce domestication have been revealed by a large-scale whole genome resequencing study [6], whether epigenetics contribute to shape the domestication traits remains unexplored.

As a conserved and pervasive epigenetic mark in most eukaryotes, DNA methylation at the C-5 position of cytosine acts as a key regulator of gene expression and modulates numerous biological processes [11–13]. In plants, DNA methylation occurs in CG, CHG, and CHH (H = A, T, or C) contexts which are maintained by different mechanisms [13]. In the model plant *Arabidopsis thaliana*, methylation of CG and CHG is maintained by METHYLTRANSFERASE 1 (MET1) [14] and CHROMOMETHYLASE 3 (CMT3) [15], respectively, while de novo CHH methylation is established by DOMAINS REARRANGED METHYLASE 2 (DRM2) through RNA-directed DNA methylation or CMT2 [13, 16]. While CHH methylation patterns exhibit developmental or tissue-specific variations in plants, CG and CHG methylations are quite stable across different vegetative tissues [17–23].

DNA methylation changes give rise to meiotically stable epialleles, which could be transmitted to offspring through natural evolution and artificial selection [24]. CG methylation inherited from parents exhibits stable transgenerational inheritance over several generations in plant genomes [25, 26]. Epialleles via DNA methylation in gene promoters have been shown to confer symmetric flower development in yellow toadflax (*Linaria vulgaris*) [27], fruit non-ripening phenotype, and change in vitamin E content in tomato [28, 29]. Despite its critical function, the role of DNA methylation in crop evolution is just beginning to be explored. Population epigenetic studies on maize [30, 31], soybean [32], and rice [33] have revealed epigenetic evolution in the long-term crop domestication processes. However, epigenome resources for vegetable crops are very limited, especially at the population level.

In this study, we report single-base resolution DNA methylomes from a natural population of 52 *Lactuca* accessions including major lettuce cultivars and wild relatives. We have defined the comprehensive DNA methylation landscape of the *Lactuca* genus and discovered DNA methylation variations that are associated with the domestication and divergence of different horticultural types of lettuce. By an integrated analysis of DNA methylomes, transcriptomes, and chromatin accessibility and interaction profiles, we reveal the effect of DNA methylation variations on influencing gene expression. Our data provide valuable epigenomic resources for vegetable improvement through epigenetic engineering.

## Results

### DNA methylation variations in the *Lactuca* genus

**Fig. 1:**
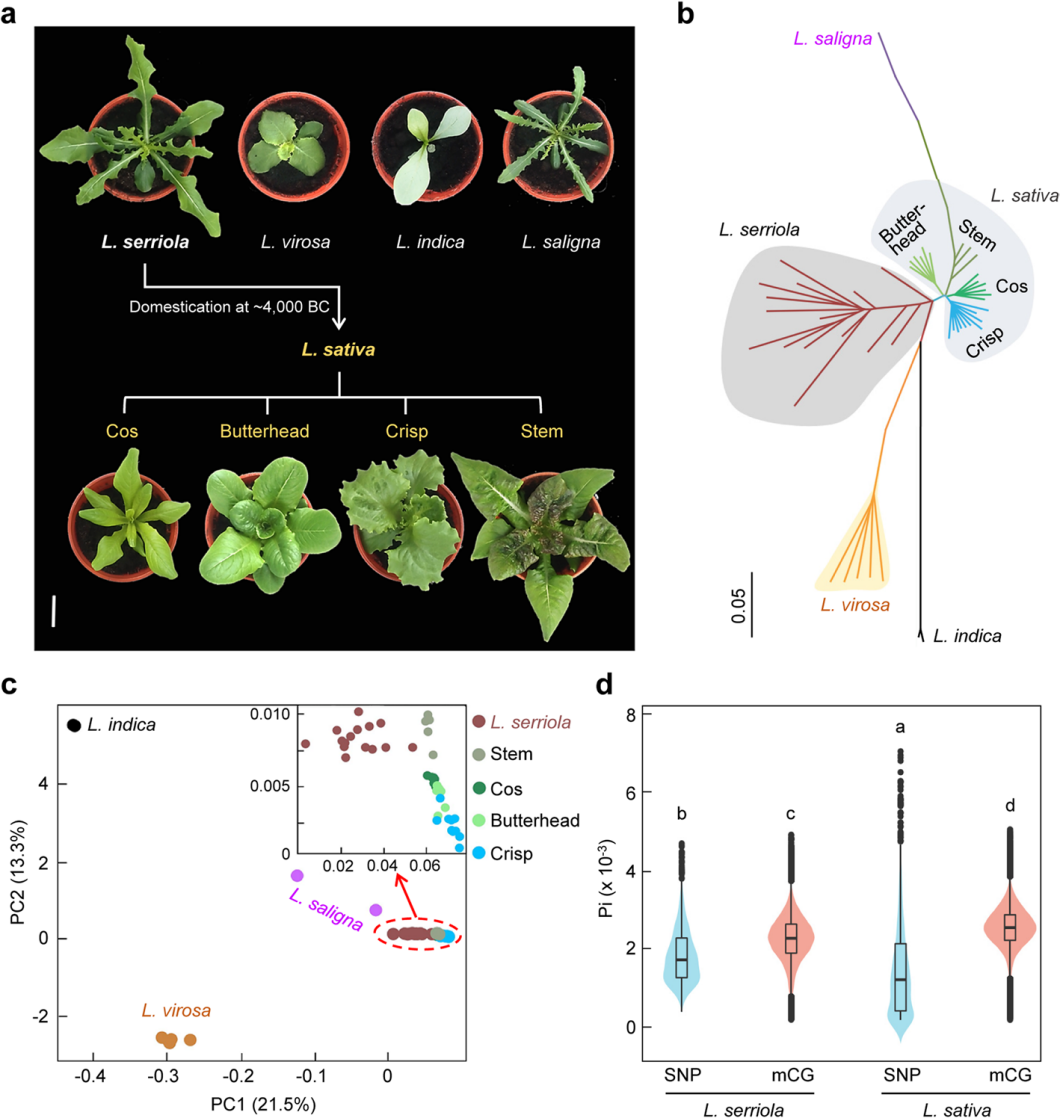
Increased CG methylation diversity during lettuce domestication. **a** Seedling morphology of the wild relative species (*L. serriola*, *L. virosa*, *L. indica*, and *L. saligna*) and cultivated lettuce including cos, butterhead, crisp, and stem lettuce at 2 weeks after planting*.* Different types of cultivated lettuce are believed to originate from a single domestication event from *L. serriola* at approximately 4000 BC [5, 6]. Scale bar, 10 cm. **b** A neighbor-joining phylogenetic tree of 52 *Lactuca* accessions based on the methylation levels of all CG loci. **c** Principal component analysis (PCA) of all *Lactuca* accessions based on CG methylation levels. **d** Diversity of CG methylation (mCG) and SNP variations of the wild lettuce (*L. serriola*) and cultivated lettuce (*L. sativa*). Different letters above the violins indicate significant differences (*P* < 0.01, two-sided Wilcoxon signed-rank test) in a pairwise comparison

To uncover the DNA methylation footprints during lettuce domestication, we generated single-base resolution DNA methylomes of 52 *Lactuca* accessions reflecting most of morphological diversity in our collection, including 28 accessions of lettuce cultivars *L. sativa* (10 crisp, 6 butterhead, 6 cos, and 6 stem lettuce) and 24 accessions of wild lettuce relatives (15 *L. serriola*, 5 *L. virosa*, 2 *L. saligna*, and 2 *L. indica*) (Fig. 1a; Additional File 1: Table S1). We performed whole-genome bisulfite sequencing (WGBS) using leaves from seedlings at 30 days after planting (DAP). Meanwhile, a total of 600 Gb whole-genome sequencing (WGS) data of these accessions were generated for genomic variant calling. To exclude the effect of genomic variations on the DNA methylation analysis, we used single nucleotide polymorphisms (SNPs) of each accession to construct their corresponding pseudo-reference genome for subsequent methylation analysis [30, 34]. Nearly 600 Gb of WGBS data of all accessions were mapped to pseudo-reference genomes. For each accession, 72 million WGBS paired-end reads (> 10 Gb) on average were generated for methylation cytosine calling. We found that the percentages of unmethylated cytosines at non-CG sites (> 17.2% for CHG and > 80.2% for CHH) were much higher than that at CG sites (< 6.89%) (Additional file 2: Fig. S1a). This observation, together with the feature of meiotically stable inheritance of CG methylation across the plant life-cycle [12, 23], promoted us to focus on analyzing the variation and evolution of CG methylation during lettuce domestication, with concurrent analysis of CHG and CHH methylation when needed.

Methylation levels at all CG sites were used to generate a neighbor-joining phylogenetic tree (Fig. 1b). This phylogenetic tree recapitulated the known evolutionary relationships of lettuce species [6], including an ancestral role of *L. serriola* for lettuce cultivars and a distant relationship between the wild relatives (*L. virosa* and *L. indica*) and cultivars (Fig. 1b). Remarkably, *L. saligna* were classified to the stem lettuce (Fig. 1b), which is different from the classifications based on SNPs (Additional file 2: Fig. S1b). Besides, phylogenetic trees based on DNA methylation levels of all CHG or CHH sites are similar to that based on CG sites (Additional file 2: Fig. S2). Moreover, the phylogenetic relationships of different lettuce groups were also supported by principal component analysis (PCA) using CG methylation levels (Fig. 1c), in which lettuce cultivars were more closely related to *L. serriola* than *L. virosa*, *L. indica*, or *L. saligna*. However, accessions of each cultivar type exhibited distribution diversities that were similar to wild lettuce accessions (Fig. 1c). In addition, the diversity (π) estimated based on CG methylation variation was higher than that based on SNPs in both lettuce cultivars *L. sativa* and the wild lettuce *L. serriola* (Fig. 1d). *L. sativa* displayed slightly higher diversity of CG methylation variation as compare with *L. serriola*, in contrast to the observed significantly lower SNP diversity in *L. sativa* than *L. serriola* (Fig. 1d) [6], suggesting that lettuce domestication is associated with rapid methylation evolution compared with genomic variation.

### A global increase of DNA methylation during lettuce domestication

**Fig. 2:**
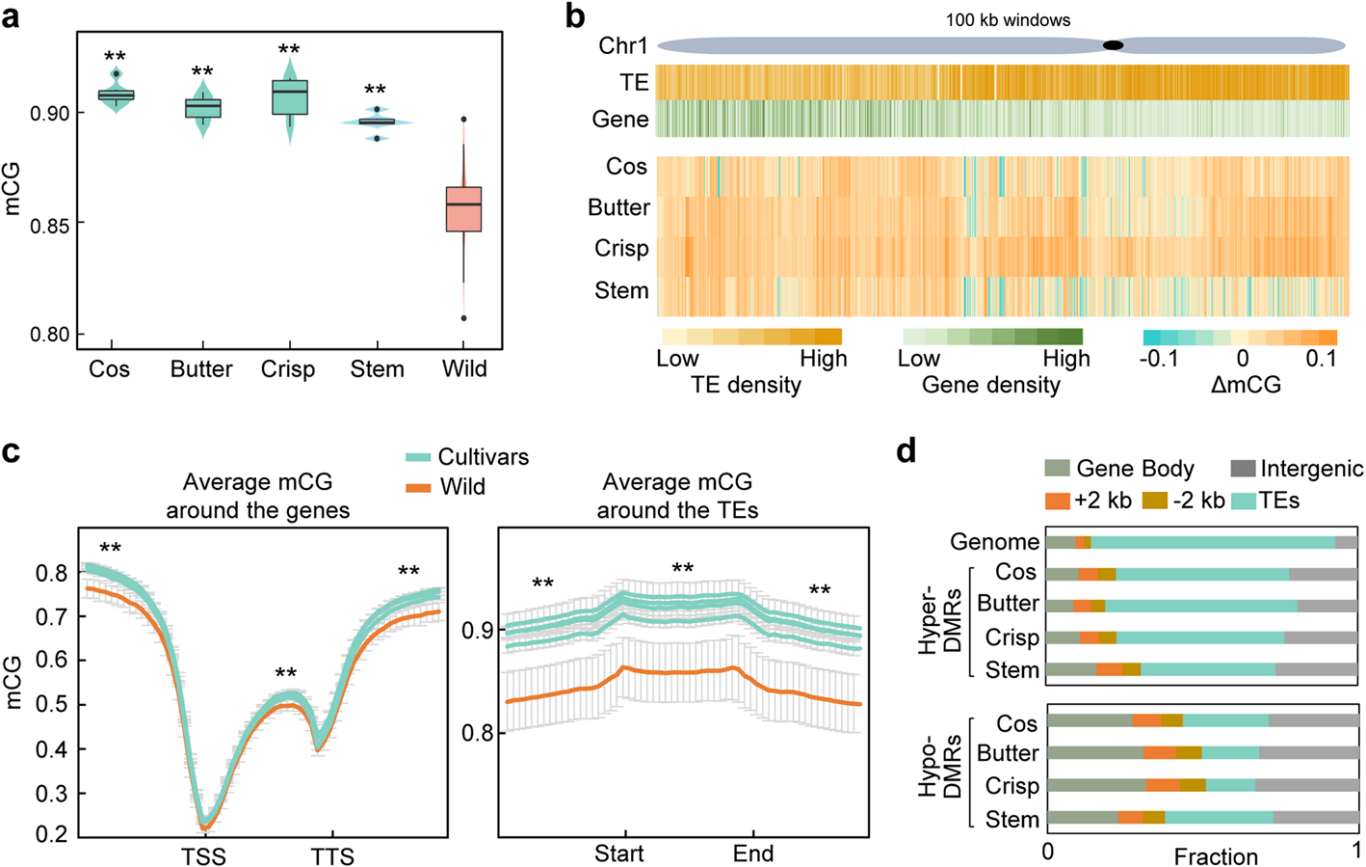
Elevated CG methylation levels during lettuce domestication. **a** Increased CG methylation levels in cultivated lettuces compared to the wild lettuce *L. serriola* (wild). Asterisks indicate significant differences of CG methylation levels (mCG) in cos, butterhead (butter), crisp, and stem lettuce as compared with the wild lettuce *L. serriola* (**P < 0.01, two-tailed paired Student’s *t*-test). **b** Heatmap of methylation level changes along chromosome 1 (Chr1) during lettuce domestication. Methylation level changes (ΔmCG) in butterhead vs. *L. serriola* (butter), cos vs. *L. serriola* (cos), crisp vs. *L. serriola* (crisp), and stem vs. *L. serriola* (stem) were calculated for each 100-kb window. Distribution of genes and TEs along Chr 1 is shown above. **c** Average CG methylation levels around genes (left) and TEs (right). TSS indicates transcription start site, TTS indicates transcription termination site. Asterisks indicate significant differences (**P < 0.01, Wilcoxon signed-rank test) of CG methylation levels in cultivars as compared with the wild lettuce *L. serriola* (wild). Cultivars include cos, butterhead, crisp and stem lettuce. See Additional file 2: Fig. S1, c and d, for the comparison of each cultivar with the wild lettuce *L. serriola*. **d** Distribution of DMRs in different genomic regions divided into gene body, +2 kb flanking region (2 kb upstream of TSS), − 2 kb flanking region (2 kb downstream of TTS), TEs, and intergenic regions excluding TEs. The average distributions of different genomic regions across the whole genome are shown as references

Modern lettuce cultivars are believed to be domesticated from the wild lettuce *L. serriola* [3, 4, 6, 7]; we thus compared methylation levels in cultivars with *L. serriola*. We found that global CG methylation levels were significantly increased in cultivated lettuce, including cos, butter, crisp, and stem lettuce, compared with *L. serriola* (Fig. 2a). Similarly, CHG and CHH methylation levels were also increased during lettuce domestication (Additional file 2: Fig. S3). CG methylation changes were enriched in euchromatic regions with protein-coding genes, especially far away from pericentromeric regions (Fig. 2b). To better understand the distribution of CG methylation changes in different regions of protein-coding genes and TEs, we calculated average methylation levels for every 100-bp interval of each gene and TE, encompassing 2-kb upstream and downstream flank regions. This analysis revealed that CG methylation levels increased greatly in the 5′ and 3′ regions and slightly in gene bodies, while they were much higher across the whole TE regions in all four horticultural types of lettuce cultivars as compared with *L. serriola* (Fig. 2c; Additional file 2: Fig. S1c,d).

To further explore the role of CG DNA methylation during lettuce domestication, we determined the differentially methylated regions (DMRs) between lettuce cultivars and the wild lettuce *L. serriola*. We identified 25,536 hyper- and 4656 hypo-DMRs in the cos group (totally 6.1 Mb, 0.24% of genome), 25,257 hyper- and 5661 hypo-DMRs in the butterhead group (totally 6.0 Mb, 0.24% of genome), 37,138 hyper- and 10,330 hypo-DMRs in the crisp group (totally 8.6 Mb, 0.34% of genome), and 7610 hyper- and 6068 hypo-DMRs in the stem group (totally 2.4 Mb, 0.09% of genome), relative to the wild lettuce *L. serriola* (Additional file 1: Table S3; Additional file 2: Fig. S4a). On average, the count of hyper-DMRs was approximately 3.5-fold higher than that of hypo-DMRs, in line with the observed global increase of methylation in lettuce cultivars (Fig. 2a). We randomly selected 36 domestication-related DMRs and most of them (34 out 36) were further confirmed by quantitative real-time PCR (qPCR; Additional file 1: Table S4), confirming the reliability of the identified DMRs. We further analyzed the distribution of DMRs across genomic features and found that hyper-DMRs were more prevalent in intergenic regions and 5′ and 3′ flanking regions of coding sequences compared to their average distributions across the whole genome (Fig. 2d). In contrast, most of the hypo-DMRs were enriched in coding sequences, 5′ and 3′ flanking regions, and intergenic regions (Fig. 2d).

### Conservation and divergence of methylation changes in cultivated lettuce

**Fig. 3:**
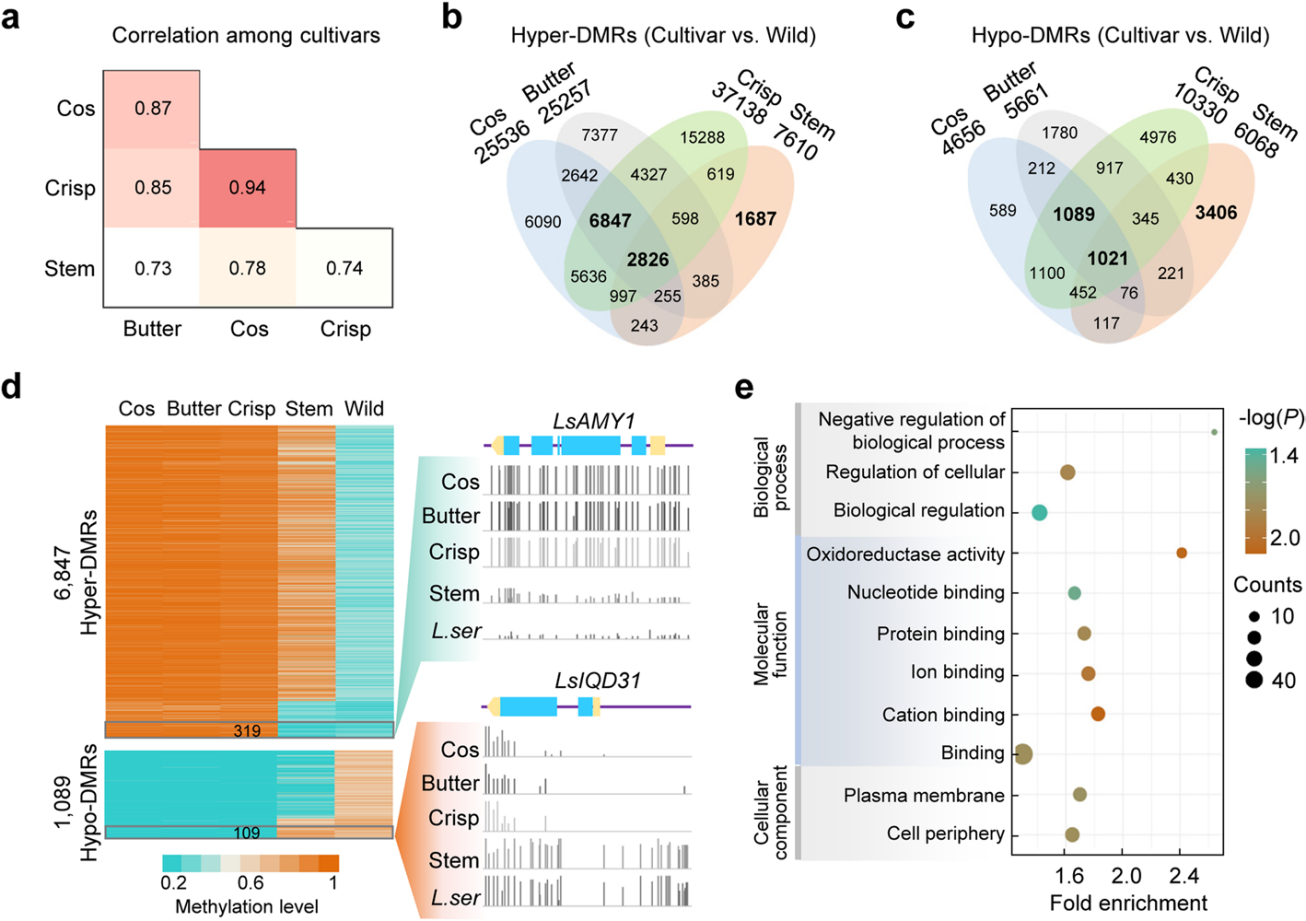
High correlation of DNA methylation changes among leafy lettuce cultivars. **a** Correction of CG methylation changes among lettuce cultivars, relative to the wild lettuce *L. serriola*. **b**, **c** Shared and unique hyper- (**b**) and hypo- (**c**) DMRs in different types of lettuce cultivars, relative to the wild lettuce *L. serriola*. **d** Heatmap showing methylation levels of type-specific hyper- and hypo-DMRs found in leafy lettuce. Leafy lettuces include cos, butterhead (butter), and crisp lettuce. Grey blocks indicate DMRs showing significant differential methylation levels between leafy lettuce and stem lettuce, representing high-fidelity leafy-specific DMRs. The left panel shows examples for a high-fidelity leafy-specific hyper-DMR across *LsAMY1* (*Lsat_1_v5_gn_6_112060*) and a high-fidelity leafy-specific hypo-DMR across *LsIQD31* (*Lsat_1_v5_gn_3_102080*). Shown above the methylation profiles are the gene structures of *LsAMY1* and *LsIQD31*, in which blue and yellow boxes indicate exons and untranslated regions, respectively, and purple lines indicate introns and other genomic regions. **e** Gene Ontology (GO) enrichment of genes associated with these high-fidelity leafy-specific DMRs

We next examined the correlation of CG methylation changes of all DMRs including both hyper- and hypo-DMRs among different types of lettuce cultivars. Notably, correlation coefficients of 0.85–0.94 were observed among pairwise comparisons of the three leafy types: cos, butterhead, and crisp lettuce (Fig. 3a). Compared to the high correlation coefficients among leaf types, lower correlation coefficients of 0.73–0.78 were found between stem lettuce and leafy types (Fig. 3a). This result indicates high degree of conservation of methylation changes among different cultivar types, which is not surprising as they are suggested to be originated from a single domestication event [5, 6]. However, significant portions of DMRs were specific for each lettuce type (Fig. 3b,c). It is noteworthy that stem and leafy (cos, butterhead, and crisp) types possessed 5093 and 7936 type-specific DMRs relative to wild lettuce, respectively (Fig. 3b,c), supporting the hypothesis that stem and leafy type lettuces are evolved independently after the initial domestication event [5].

Nevertheless, further analysis on these type-specific DMRs found in leafy lettuce revealed that stem lettuce also displayed consistent but insignificant changes in methylation levels on most of these DMRs relative to the wild lettuce (Fig. 3d). In contrast, 319 (4.7%) hyper-DMRs and 109 (10%) hypo-DMRs of these type-specific DMRs found in leafy lettuce exhibited significant methylation changes as compared to stem lettuce and were exclusively detected in leafy lettuce, which were called high-fidelity leafy-specific DMRs (Fig. 3d). For examples, *LsAMY1* (*Lsat_1_v5_gn_6_112060*), homolog to *AtAMY1* involved in starch mobilization and response to biotic and abiotic stress [35–37], showed high methylation levels across its gene region only in leafy lettuce (Fig. 3d). On the contrary, *LsIQD31* (*Lsat_1_v5_gn_3_102080*), homolog to *AtIQD31* involved in plant development [38], displayed low methylation levels particularly in leafy lettuce (Fig. 3d). These high-fidelity leafy-specific DMRs tended to occur in flanking sequences of genes and intergenic regions (Additional file 2: Fig. S4b). Genes associated with these DMRs were enriched in regulation processes including negative regulation of biological process, biological regulation, and regulation of cellular process (Fig. 3e; Additional file 1: Tables S5 and S6).

### Independent evolution of stem lettuce

**Fig. 4:**
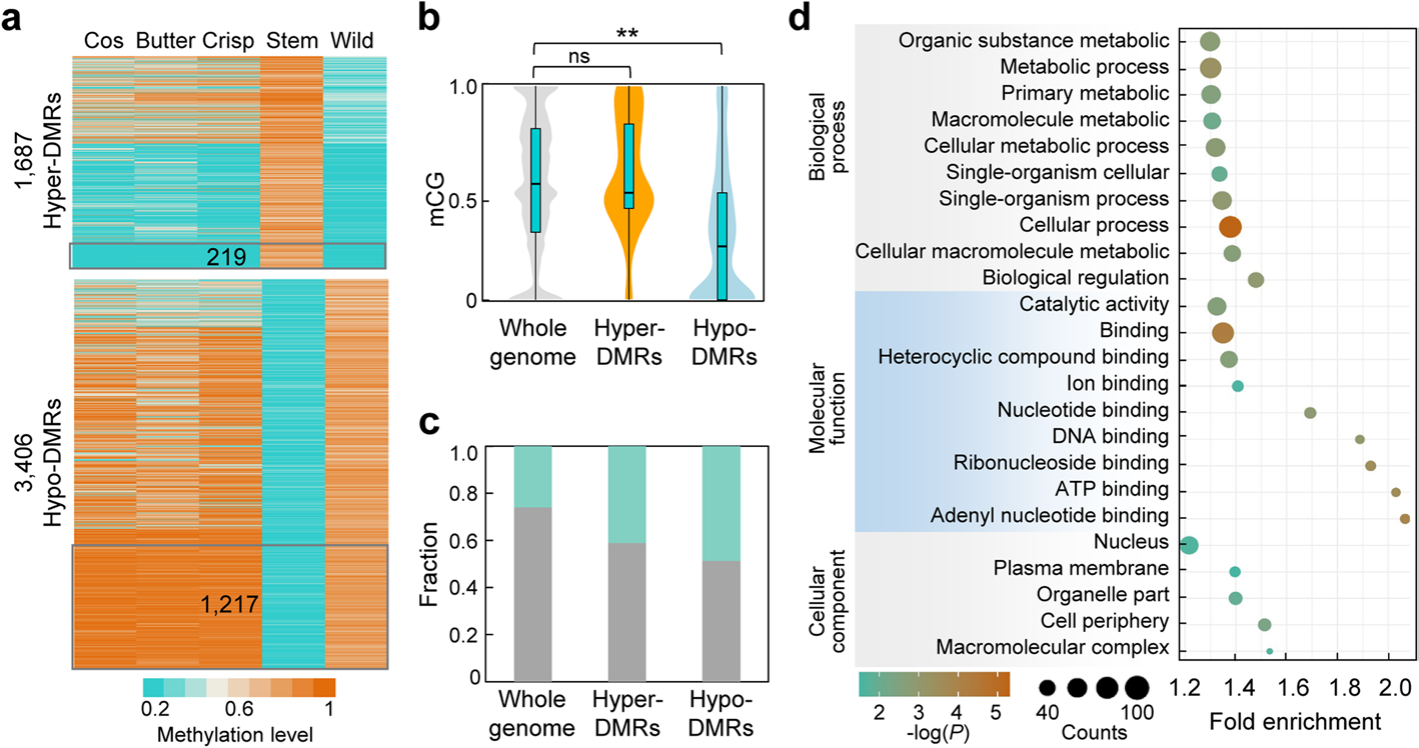
Independently altered DNA methylation in stem lettuce. **a** Heatmap showing methylation levels of the stem lettuce specific hyper- and hypo-DMRs, relative to the wild lettuce *L. serriola*. DMRs in the grey blocks show significant differences between leafy type and stem lettuce, representing high-fidelity stem-specific DMRs. **b** Methylation levels of the high-fidelity stem-specific DMRs in the wild lettuce *L. saligna*. Asterisks and ns indicate significant difference (**P < 0.01, Wilcoxon signed-rank test) and no statistical difference (*P* ≥ 0.01, Wilcoxon signed-rank test), respectively. **c** Enrichment of regions with the same SNPs in *L. saligna* and stem lettuce observed in high-fidelity stem-specific DMRs. Blue bars indicate fractions of regions with the same SNPs in stem lettuce and *L. saligna*, but different from other *Lactuca* species, including cos, butter, and crisp lettuce, *L. serriola*, *L. virosa*, and *L. indica*. **d** GO enrichment of genes associated with the high-fidelity stem-specific DMRs

We then further analyzed the type-specific DMRs found in stem lettuce (Fig. 3b,c) and found that most of them showed similar methylation levels in leafy lettuce as compared to the wild lettuce *L. serriola* (Fig. 4a). Among them, 219 (13.0%) hyper-DMRs and 1217 (35.7%) hypo-DMRs exhibited significant methylation changes compared to leafy lettuce (Fig. 4a), which are referred hereafter as high-fidelity stem-specific DMRs. This result implies that evolution of stem lettuce, characterized by many distinct DMRs, is eccentric and independent of the leafy lettuce. Interestingly, the wild relative *L. saligna* was clustered into a subgroup of stem lettuce in the phylogenetic tree (Fig. 1b) despite their phenotypic divergence (Fig. 1a) and huge nucleotide diversity evident from the phylogenetic tree based on SNPs (Additional file 2: Fig. S1b). We thus further analyzed the methylation levels of high-fidelity stem-specific DMRs in *L. saligna*. We found that *L. saligna* showed low methylation levels on the hypo-DMRs compared to the average methylation level across the whole genome but no significant change on the hyper-DMRs (Fig. 4b). In contrast, two other wild relatives *L. virosa* and *L. indica* showed opposite changes with low methylation levels on the hyper-DMRs and high methylation levels on the hypo-DMRs (Additional file 2: Fig. S5a,b). These results imply the possible existence of a unique epigenetic evolution mechanism in stem lettuce in relation to *L. saligna*.

To uncover the origin of these high-fidelity stem-specific DMRs, we utilized global SNPs to identify windows where these DMRs exhibited the same genotype as stem lettuce in comparison with only one species of wild relatives but different from other wild relatives and leafy lettuce. In the global 9,998,549 windows for DMR identification, 26% of these windows exhibited a unique genotype that was shared by stem lettuce and *L. saligna*, which was more than 8-fold higher than other wild relatives (3.1% for *L. serriola*, 0.13% for *L. virosa*, and 0.08% for *L. indica*) (Additional file 2: Fig. S5c). Moreover, 41% and 48% of high-fidelity stem-specific hyper-DMRs and hypo-DMRs, respectively, overlapped with the identified windows where stem lettuce shares same genotypes as *L. saligna* (Fig. 4c). This result indicates that *L. saligna* could be the potential origin for the distinctive methylation variations in stem lettuce, thereby distinguishing it from leafy lettuce.

In addition, the high-fidelity stem-specific DMRs tended to occur in flanking sequences of genes as well as intergenic regions (Additional file 2: Fig. S5d). Genes associated with these DMRs were enriched into metabolic processes including organic substance metabolic, primary metabolic, and macromolecule metabolic (Fig. 4d; Additional file 1: Tables S7 and S8), implying a distinct selection on the metabolic processes during diversification of stem lettuce.

### Domestication-induced DMRs are associated with stress response

**Fig. 5:**
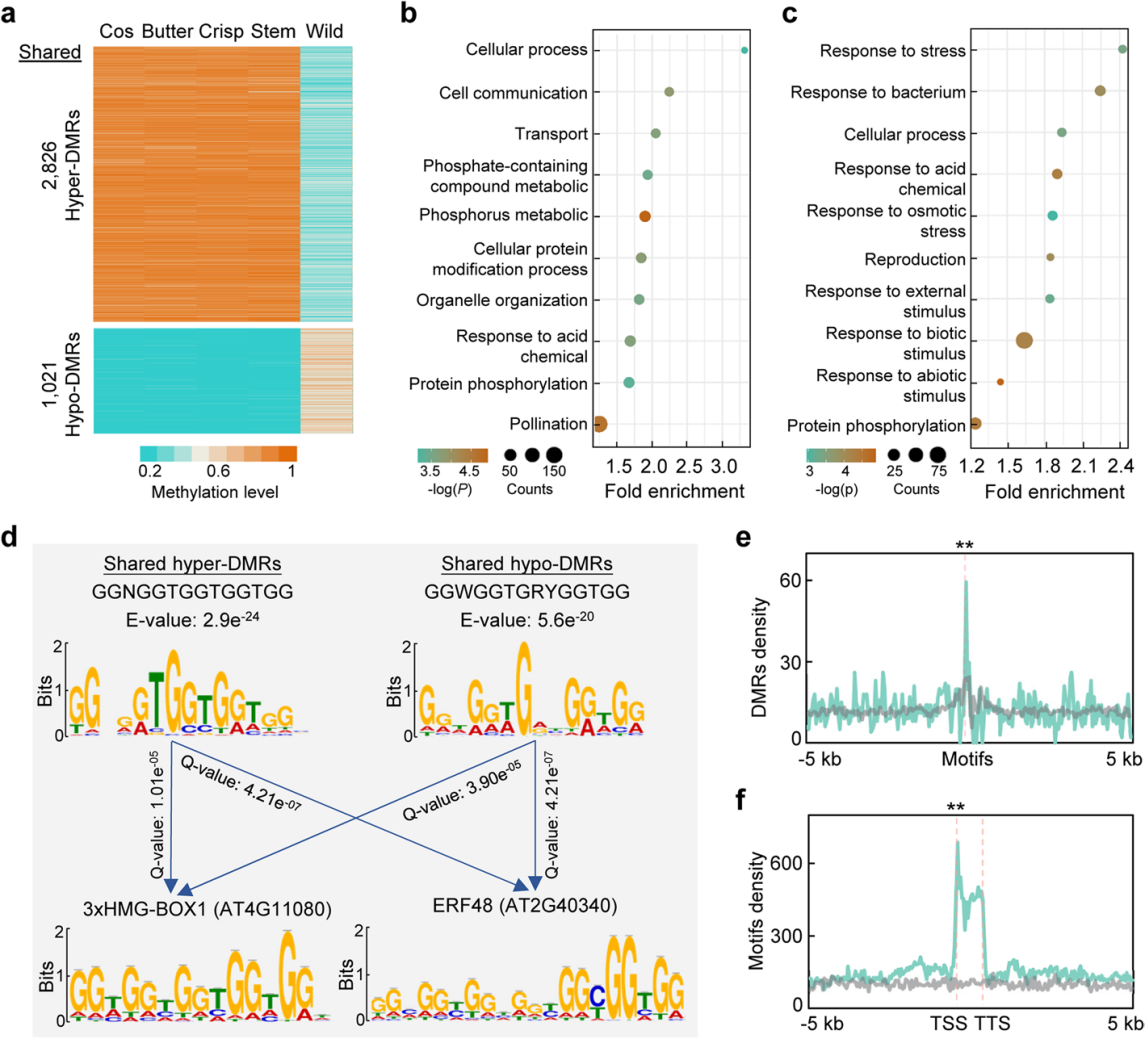
Shared domestication-induced DMRs of lettuce cultivars. **a** Heatmap showing methylation levels of shared hyper- and hypo-DMRs of lettuce cultivars. **b**, **c** GO enrichment of genes associated with shared hyper- (**b**) and hypo-DMRs (**c**). The plots show 10 top-scoring biological processes. **d** DNA motifs enriched in the shared domestication-induced DMRs (upper panels) are similar to the motifs of binding sites of 3XHMG-BOX1 and ERF48 in published dataset (lower panels). *E*-value (*e*) indicates an estimate of the expected number of motifs by the MEME, while *Q*-value (*q*) indicates the probability that a random motif has an optimal alignment as the target motif. **e** Density of shared DMRs within 10 kb of ERF48 binding motif. The grey line indicates random genomic regions. Asterisk indicates significant difference (**P < 0.01, Wilcoxon signed-rank test). **f** Density of ERF48 binding motif within 10 kb of genes. The grey line indicates random genomic regions. Asterisk indicates significant difference (**P < 0.01, Wilcoxon signed-rank test)

In addition to type-specific DMRs, it was noteworthy that 2826 (7.61–37.1%) of hyper-DMRs and 1021 (9.88–21.9%) of hypo-DMRs were shared between leafy and stem lettuce (Figs. 3b, c and 5a). These shared domestication-induced DMRs accounted for 0.027% (0.698 Mb) of lettuce genomes which was significantly higher than that expected by chance (*P* = 0, hypergeometric test) and were called shared DMRs. These DMRs tended to occur in flanking sequences of genes and intergenic regions (Additional file 2: Fig. S6a). Genes associated with shared hyper-DMRs were enriched in cellular process, cell communication process, transport process, phosphate-containing compound metabolic process, response to acid chemical process, etc. (Fig. 5b; Additional file 1: Tables S9 and S10). In contrast, genes associated with domestication-induced shared hypo-DMRs were mainly enriched in biotic and abiotic stress response pathways including response to bacterium, response to acid chemical, and response to osmotic stress (Fig. 5c; Additional file 1: Tables S9 and S11), which is consistent with commonly speculated growth-defense trade-off during the process of crop domestication. In addition, we observed consistent changes of non-CG (CHG and CHH) methylation levels with CG methylation in these shared-DMRs between cultivated and wild lettuce (Additional file 2: Fig. S7), implying conserved changes of DNA methylation on different cytosine contexts during lettuce domestication. Notably, shared hypo-DMRs with very low CG methylation levels also contained low levels of CHG and CHH methylation in cultivated lettuce (Additional file 2: Fig. S7a).

As shared DMRs frequently occurred around genes (Additional file 2: Fig. S6a), we further performed MEME motif analysis [39] and found that shared hyper-DMRs possessed a top-scoring motif of GGNGGTGGTGGTGG (*E* = 2.9 × 10^−24^), while shared hypo-DMRs had a top-scoring motif of GGWGGTGRYGGTGG (*E* = 5.6 × 10^−20^) (Fig. 5d). These two motifs resembled the binding sites of 3xHMG-BOX1 and AtERF48 [40]. 3xHMG-BOX1 belongs to a plant-specific family of DNA-binding proteins that interact with mitotic and meiotic chromosomes [41], while AtERF48, member of APETALA2/ethylene response factor (ERF) transcription factor family, is responsible for modulating stress responses [42–44]. Remarkably, shared DMRs showed enrichment in the binding motif of ERF48 (Fig. 5e), and this motif tended to enrich in the gene body, particularly at the TSS (Fig. 5f). We then further identified the target genes of LsERF48 (Lsat_1_v5_gn_8_13141) through DNA affinity purification sequencing (DAP-seq) in lettuce (Additional file 1: Table S12). A GGWGGTGGTGG motif was found to be enriched in the binding sites of LsERF48 (Additional file 2: Fig. S8a), which resembled the motifs found in AtERF48 binding sites and shared DMRs (Fig. 5d). Consistent with the motif distribution observed in shared DMRs (Fig. 5e), LsERF48 binding sites were also more prevalent in the gene body (Additional file 2: Fig. S8b). Interestingly, LsERF48 target genes were enriched in stress response (Additional file 2: Fig. S8c), implying its possible role in modulating stress response in lettuce. Remarkably, 38 out of 671 genes associated with shared DMRs were also identified as LsERF48 targets (Additional file 1: Table S12). In addition, *35S:LsERF48-GFP*, but not the *35S:GFP* control, reduces reactive oxygen species (ROS) accumulation in response to salt stress in *N. benthamiana* (Additional file 2: Fig. S9), supporting the role of LsERF48 in modulating stress response. Together, these results imply domestication-induced DNA methylation variations in lettuce may play a potential role in regulating stress response.

### Domestication-induced DNA methylation variations influence gene expression through *cis*- or trans-acting effects

**Fig. 6:**
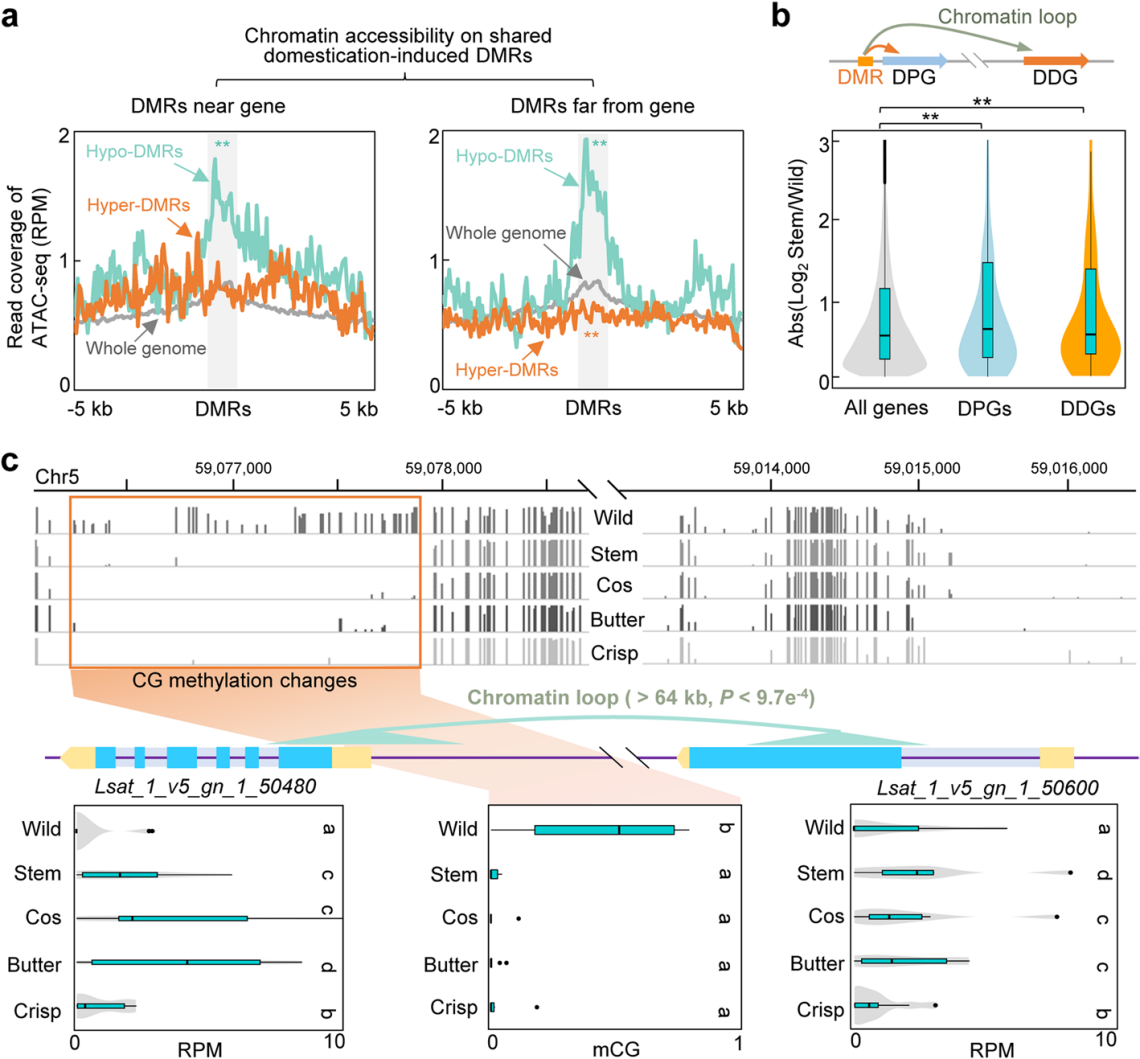
Domestication-induced DNA methylation changes contribute to gene expression changes through *cis*- or *trans*-acting effects. **a** Metaplots showing chromatin accessibility in the shared domestication-induced DMRs of lettuce cultivars, located near the genes (left panel) and far from genes (right panel). Asterisks indicate significant differences (**P < 0.01, Wilcoxon signed-rank test) of the chromatin accessibility in DMRs compared with the whole genome. **b** Increased gene expression changes (absolute values) between stem lettuce and the wild lettuce *L. serriola* in DMR-associated proximal genes (DPGs) and distal genes (DDGs) compared with all genes. Asterisks indicate significant differences (**P < 0.01, Wilcoxon signed-rank test). **c** An example showing methylation changes (Chr1: 59,076,400–59,077,800; upper panel and middle box plot in the lower panel) related to the proximal gene (DPG: *Lsat_1_v5_gn_1_50480*; right box plot in the lower panel) and the distal gene (DDG: *Lsat_1_v5_gn_1_50600*; left box plot in the lower panel) that are in one chromatin loop with more than 64 kb physical distance. The middle panel shows the gene structures of these genes, in which blue and yellow boxes indicate exons and untranslated regions, respectively, and purple lines indicate introns and other genomic regions. Different letters on the boxes indicate significant differences (*P* < 0.01, Wilcoxon signed-rank test) in a pairwise comparison

As the majority of DNA methylation signatures are related to chromatin accessibility in plants [23, 45], we proceeded to examine whether domestication-induced DNA methylation variations affect chromatin accessibility in lettuce. To this end, we analyzed the ATAC-seq data obtained from stem lettuce [46] to investigate the chromatin accessibility patterns on the shared DMRs. We classified these shared DMRs based on their distance to genes: DMRs near genes that were located in 2 kb flanking sequences (1124/3847, 29%) and DMRs far from genes (2723/3847, 71%). Interestingly, we found that chromatin accessibility was significantly increased at shared hypo-DMRs near genes, but insignificantly decreased at shared hyper-DMRs near genes (Fig. 6a). Moreover, shared hypo-DMRs far away from genes showed higher levels of chromatin accessibility, whereas shared hyper-DMRs far away from genes showed lower levels of chromatin accessibility (Fig. 6a). To further explore whether DNA methylation changes influences chromatin accessibility during lettuce domestication, we assessed chromatin accessibility in the wild lettuce *L. serriola* through ATAC-seq. Notably, we observed high levels of chromatin accessibility in wild lettuce in shared hyper-DMRs near genes and far away from genes (Additional file 2: Fig. S10), where wild lettuce possessed low DNA methylation levels (Fig. 5a). This observation, together with the relatively low chromatin accessibility in shared hyper-DMRs in stem lettuce that possessed high DNA methylations in these regions (Fig. 6a), indicate that domestication is associated with an increase in DNA methylation alongside a decrease in chromatin accessibility in stem lettuce compared to wild lettuce.

To further examine whether DNA methylation changes influence gene expression, we identified 673 DMR-associated proximal genes (DPGs) that had shared DMRs located within 2 kb flanking sequences and detected 149 DMR-associated distal genes (DDGs) that interact with shared DMRs via long-range chromatin loops through reanalyzing the published Hi-C datasets of stem lettuce (Fig. 6b; Additional file 1: Table S13) [46, 47]. We also conducted RNA-seq analysis of all our accessions of wild and cultivated lettuce (Additional file 1: Table S2). DNA methylation changes significantly induced expression changes of both DPGs and DDGs in stem lettuce (Fig. 6b; Additional file 2: Fig. S11). For example, a shared hypo-DMR in Chr 1 (59,076,400–59,077,800) was associated with increased expression of its neighboring gene *Lsat_1_v5_gn_1_50480*, a homolog of *AT2G36540* with unknown function, as well as a distal gene *Lsat_1_v5_gn_1_50600*, a homolog of *AT5G04550* related to abiotic stress responses [48], that is located at ~ 64 kb downstream of this DMR (*P* value < 9.7 × 10^−4^ for the interaction loop) (Fig. 6c). The expression changes of *Lsat_1_v5_gn_1_50480* and *Lsat_1_v5_gn_1_50600* in cultivated lettuce compared with wild lettuce were further verified by qPCR (Additional file 2: Fig. S12). Inhibition of DNA methylation by 5-azacytidine (5-AzaC) in the wild lettuce (*L. serriola*) seedlings resulted in a reduction of DNA methylation levels in Chr1: 59,076,400–59,077,800 but elevated expression levels of *Lsat_1_v5_gn_1_50480* and *Lsat_1_v5_gn_1_50600* (Additional file 2: Fig. S13). Similar to stem lettuce, DNA methylation variations were also associated with significant expression changes of DPGs and DDGs in cos lettuce (Additional file 2: Fig. S6b). In addition, we also found that DNA methylation changes were associated with increased expression of DPGs in butterhead and crisp lettuce (Additional file 2: Fig. S6b-d). These results indicate that variations in DNA methylation may affect chromatin accessibility and hence alter expression levels of the associated proximal and distal genes.

## Discussion

As an essential and pervasive epigenetic mark in most eukaryotes, DNA methylation controls multiple biological and physiological processes [13, 49]. Several recent population epigenetic studies suggest roles of epigenetics evolution in the long-term domestication processes of several crops including maize [30, 31], soybean [32], cotton [50], and rice [33]. In this study, we have defined the DNA methylation landscape in a natural population of 52 *Lactuca* accessions including major lettuce cultivars and wild relatives and uncovered extensive DNA methylation variation during lettuce domestication and divergence.

Lettuce belongs to the core group of crops that are cultivated worldwide and represents one of the earliest domesticated vegetable crops (~ 6000 years ago) [2, 6]. Lettuce cultivars are believed to be derived from *L. serriola* in a single domestication event [5]. It has been estimated that stem and leafy type lettuce (butterhead, crisp and cos) were diverged from the ancestral cultivated lettuce at ~1922 and ~500 years ago [5]. Our phylogenetic analysis based on DNA methylation of the *Lactuca* genus further supports the ancestral role of *L. serriola* for lettuce cultivars but also indicates an interesting relationship between the wild relative *L. saligna* and stem lettuce. Transfer of genetic material between crops and their wild relatives through spontaneous hybridization and subsequent backcrossing, known as introgression, widely occurred and has been suggested to promote crop domestication and diversification [51–54]. Our analysis has revealed that 41–48% of the high-fidelity stem-specific DMRs are related to the introgression of *L. saligna*, greatly higher compared to other wild relatives *L. serriola*, *L. virosa*, and *L. indica* (Fig. 4c; Additional file: Fig. S5c). Among the noticeably increased fraction of *L. saligna*-related DMRs, the hypo-methylations of *L. saligna* could potentially exhibit transgenerational inherence during stem lettuce evolution and improvement, while most of hyper-methylations could be unstable (Fig. 4b). We envisage that these unstable hyper-methylations might undergo functional demethylation and/or be influenced by the dilution of factors for maintaining methylation, as previously reported [34]. These methylation variations are likely a response to artificial domestication. Our result implies that although wild relatives are commonly used to introduce advanced traits such as high production, rich nutrition, and resistances to abiotic and/or biotic stimulus [6, 55], epigenetics may play an important role in determining the fate of functionalization during genetic introgression.

It has been hypothesized that stem and leafy (butterhead, crisp, and cos) types of lettuce are evolved independently after the initial domestication in the domestication center [5]. We have uncovered abundant DNA methylation variations that are specifically associated with leafy and stem types, in addition to those commonly associated with both types. Interestingly, these variations are found to be enriched in different GO categories, likely contributing to morphological domestication and diversification of lettuce. Moreover, these variations, together with the dataset showing expression levels of the genes related to the stem lettuce-specific DMRs in different types of cultivated lettuce and the wild lettuce *L. serriola* (Additional file 1: Table S14), serve as valuable resources for further functional genomic research on lettuce divergence.

Although abundant genetic variations during lettuce domestication have been profiled by a large-scale whole genome resequencing study [6], there are few examples of genes that have been shown to be directly linked to lettuce domestication. Characterized lettuce genes that are possibly involved in lettuce domestication include lettuce *APETALA2* regulating seed shape [56], *LsKN1* controlling leafy head development [57], and *LsNRL4* regulating photosynthesis and leaf angles [58]. Unfortunately, these genes do not contain domestication-induced DMRs. Nevertheless, we have identified hundreds of genes that are associated with these domestication-induced DMRs, which will serve as valuable resources to facilitate future functional genomic studies in investigating domestication genes in lettuce. Furthermore, it has been suggested that the domestication process is driven significantly by the adaptation of stress tolerance to human-provided environments and conditions, leading to considerable changes in morphology, behavior, and physiology, and notably, reduced stress resistance is a common trait observed in many crop domestications [59, 60]. In this study, the enrichment of stress response genes has been found in the genes associated with the shared hypo-DMRs (Fig. 5c), and interestingly, the shared DMRs show enrichment in the binding motif of ERF48 that is possibly involved in modulating stress response (Fig. 5e; Additional file 2: Fig. S9). Stress conditions has been shown to induce DNA methylation in plants [61–63], and cultivated crops grown in human-manipulated environments are often accompanied by tradeoffs in stress resistance to promote growth and yield [64, 65]. It is possible that DNA methylation related to stress response may passively decrease during lettuce domestication. Differential responses to pathogens have been observed in lettuce cultivars and wild relatives [9]; it would be intriguing to further explore the involvement of the identified epialleles in responses to various stress conditions in lettuce.

Long-range interactions between genomic elements are critical for many cellular processes through regulation of gene expression [47, 66, 67]. For example, in maize, a distal enhancer *KERNEL ROW NUMBER4* (*KRN4*) located at more than 30 kb downstream from the *UNBRANCHED3* (*UB3*) gene could spatially interact with the *UB3* promoter to affect *UB3* expression and hence regulate inflorescence development [68]. Likewise, our analysis has revealed that DNA methylation variations influence not only gene expression changes in their proximal genes but also their distal genes involved in long-range chromatin loops (Fig. 6b) and that expression of shared hypo-DMR-associated genes is significantly increased, but not vice versa for hyper-DMR-associated genes (Additional file 2: Fig. S14). However, we have observed some discrepancies among different lettuce cultivars. DNA methylation variations influence expression levels of both proximal and distal genes in stem and cos lettuce but mainly affect proximal genes in butterhead and crisp lettuce (Additional file 2: Fig. S6c,d). This could be due to the differences in the chromatin loop profiles in different lettuce cultivars. It has been suggested that chromatin structures are dynamically altered during soybean polyploidization, diploidization, and domestication [69]. Vast chromatin loops between genes and genes and those between gene and distal regulator elements are generated or eliminated during soybean domestication [69]. Thus, further mapping the chromatin interaction profiles in the *Lactuca* genus will be necessary to better understand roles of epigenetic evolution in influencing gene expression and morphological changes during lettuce domestication.

## Conclusions

In summary, our study reveals epigenome variations among major cultivated lettuce and wild relatives, providing important insights into epigenetic evolution in gene regulation during lettuce domestication and divergence. These identified epialleles serve as valuable resources for facilitating future functional genomic research into lettuce domestication and divergence as well as for epigenetic breeding to improve lettuce production and stress resistance for sustainable agriculture.

## Methods

### Plant materials and growth conditions

Lettuce seeds were obtained from the Center for Genetic Resources, the Netherlands (http://www.wageningenur.nl/) (Additional file 1: Table S1). Seeds were surface sterilized with 10% sodium hypochlorite and grown on soil in a growth chamber with 16 h light/8 h dark at 24°C (day)/22 °C (night). The third pair of leaves at 30 days after planting (30 DAP) was collected at ZT4 (Zeitgeber time) for isolating genomic DNA and total RNA for library construction.

### Library construction for MethylC-seq and DNA-seq

Genomic DNA was isolated from the leaf samples using the cetyltrimethylammonium bromide (CTAB) method [70]. After removing RNA with RNase A (NEB), genomic DNA (about 3 μg) was fragmented into 300–500 bp long, end-repaired, and 3′-end adenylated followed by ligation of the methylated adapter (AITbiotech) according to the protocol of NEBNext® Ultra™ II DNA Library Prep Kit for Illumina® (NEB). For MethylC-seq, around 1 μg of adapter-ligated DNA fragments was treated with bisulfite using the Zymo EZ DNA Methylation-Gold^TM^ kit (Zymo Research), followed by a 10-cycle PCR amplification with Q5U® HiFi Hot Start DNA Polymerase (NEB). For DNA-seq libraries, the rest (around 1 μg) of the above adapter-ligated DNA fragments was amplified by a 6-cycle PCR amplification with Q5® HiFi Hot Start DNA Polymerase (NEB). After purification with VAHTSTM DNA Clean Beads (Vazyme), both MethylC-seq and DNA-seq libraries were sequenced on a NovaSeq platform (Illumina), generating 150 bp paired-end reads.

### SNP calling

After filtering the raw reads of DNA-seq libraries with fastp [71], clean reads were mapped to the lettuce reference genome (Salinas_v8, https://phytozome-next.jgi.doe.gov/info/Lsativa_V8) [72] using BWA (v0.7.15) with default parameters [73]. Uniquely mapped reads were extracted, and potential PCR duplicates were removed using Picard-tools (version 2.0.1). The remaining reads were used for variant detection using Genome Analysis Toolkit (GATK, version 3.5.0) [74]. SNPs and Indels were separated using the GATK “SelectVariants” function. To reduce the variant discovery rate, the SNP calls were filtered according to the following threshold: QD (quality by depth) < 10.0 || MQ (mapping quality) < 20.0 || FS (fisher strand) > 30.0 || SOR (symmetric odds ratio) > 3.0 || MQRankSum (rank sum test for mapping quality) < -2.5 || ReadPosRankSum (rank sum test for read position bias) < -3.5 || DP (read depth) <5 || DP > 100.0. The remaining SNPs were used for further analyses.

### Single cytosine methylation calling and population parameter (Pi, π)

After filtering the raw reads of MethylC-seq libraries with fastp [71], the clean reads of each accession were mapped to the corresponding pseudo reference genome [30, 34], in which the lettuce reference genome (Salinas_v8) sequences were replaced by the corresponding nucleotides of each accession at the SNPs loci [75, 76], using Bismark (v0.15.0) with options (--score_min L,0,-0.2 -X 1000). Bisulfite conversion of all libraries was evaluated using the chloroplast genome as a control. The average conversion rate of 98.5% indicates a high level of reproducibility. To reduce clonal bias, the reads mapped to the same sites were collapsed into a single consensus read for calling methylation level on each cytosine site covered by at least three uniquely mapped reads.

To distinguish unmethylated cytosines in the CG contexts, we analyzed methylation level using the mixtools [77] under “normalmixEM” and set the thresholds as follows: unmethylation (≤ 0.1), heterozygous methylation (> 0.1 and < 0.9), methylation (≥ 0.9). To calculate the diversity of DNA methylation in lettuce population, we used methylation haplotype (meplotype) by converting the methylation status of each cytosine to nucleobase based on the threshold of the methylation level: methylation level ≤ 0.1 marked as “TT” representing unmethylated cytosine, methylation level within 0.1–0.9 marked as “CT” representing partially methylated cytosine, and methylation level ≥ 0.9 marked as “CC” representing methylated cytosine [78]. The meplotype and SNPs after removing variants with > 40% missing calls and minor allele frequency (MAF) < 0.05 were used to calculate population parameter (π) using VCFtools (0.1.13) [79] in 100-kb windows with 10-kb steps across the genome.

### Identification of differentially methylated regions (DMRs)

To identify DMRs among the lettuce population including the wild lettuce *L. serriola* population and each horticultural types of cultivated lettuce population, accessions from each population were considered as repeats and cytosine (CG) sites detected in at least 3 accessions from each population which were considered as effective cytosine sites. The genomic windows containing eight consecutive effective cytosine sites (< 500 bp) were used to identify DMR candidate regions using DMRfinder (v0.3) [80], in conjunction with R language (V4.3.1) for statistical tests and adjusting *P*-values. Within these candidate regions, CG DMRs between two populations were determined by applying cut-off values for average methylation level differences (> 0.4) and a corrected false discovery rate (FDR < 0.05). The FDR was calculated by adjusting the P-values (obtained from ANOVA tests) of pairwise comparisons using the Benjamini-Hochberg method.

### Analysis of DMRs using qPCR

The DMRs were validated using qPCR with the digested methylated genomic DNA as previously reported [81]. Briefly, genomic DNA (1 μg) was digested for 16 h using MspJI (NEB), with mock treatments performed by substituting glycerol for MspJI. qPCR was performed using Taq Pro Universal SYBR Green Master Mix (Vazyme) on the CFX Opus 384 Real-Time PCR System (Bio-Rad). Relative DNA methylation levels were assessed by calculating the difference between mock Ct and digested Ct values, and a larger differential Ct value represents a higher methylation level. The primers for the randomly selected 36 DMRs are listed in Additional file 1: Table S15.

### Library construction and analysis of DAP-seq

DAP-seq libraries were constructed as previously reported [82]. To generated adapter-ligated DNA fragments, the genomic DNA (~3 μg) was fragmented into 300–500 bp long, end-repaired, and 3′-end adenylated followed by ligation of the adapter (AITbiotech) according to the protocol of NEBNext® Ultra™ II DNA Library Prep Kit for Illumina® (NEB). The full-length coding sequence of *LsERF48* was amplified with P1F and P1R (Additional file 1: Table S14) and cloned into the pCAMBIA1300-GFP vector. The resulting pCAMBIA1300-*LsERF48*-GFP plasmid was added to the TNT® SP6 Coupled Wheat Germ Extract System (Promega) to synthesize the LsERF48-GFP protein. The LsERF48-GFP protein was then immobilized on the GFP-Trap® Agarose Beads (ChromoTek) and subsequently incubated with 100 ng of adapter-ligated DNA fragments. After extensive washing, the eluted DNA was amplified through 20 cycles of PCR using Q5 HiFi HotStart DNA Polymerase (NEB). Following purification, the DAP-seq libraries were sequenced on the NovaSeq platform (Illumina), generating 150 bp paired-end reads.

After filtering the raw reads of DAP-seq with fastp [71], clean reads were mapped to the lettuce reference genome (Salinas_v8) using BWA (v0.7.15) [73]. Uniquely mapped reads were extracted, followed by removing potential PCR duplicates using Picard-tools (version 2.0.1). The remaining paired reads were then used for peak calling using MACS2 (2.2.6). Total peaks were used to perform DNA motifs analysis using MEME (5.1.0).

### ATAC-seq library construction

Construction of ATAC-seq libraries was performed using the ATAC-seq kits (Activemotif) according to the manufacturer’s instructions. DNA was recovered by MinElute PCR Purification Kit (Qiagen) and amplified through 10 cycles of PCR. Libraries were sequenced on the NovaSeq platform (Illumina) to generate 150 bp paired-end reads.

### Analysis of ATAC-seq and Hi-C seq datasets

The ATAC-seq data and published ATAC-seq datasets downloaded from https://www.lettucegdb.com/data/ATAC-Seq/ (Additional file 1: Table S2) were filtered with fastp [71]. Clean reads were mapped to the lettuce reference genome (Salinas_v8) using BWA (v0.7.15) [73]. Uniquely mapped reads were extracted, followed by removing potential PCR duplicates using Picard-tools (version 2.0.1). Using the remaining paired reads, read counts in 50-bp bins across the whole genome were normalized as reads per million mapped reads (RPM). To reveal changes in chromatin accessibility between DMRs and random genomic regions, the genome was divided into windows to match the average length of DMRs, and subsequently the same numbers of windows were randomly selected for comparison with DMRs.

Hi-C data were downloaded from BIG Data Center (https://bigd.big.ac.cn/gsa/index.jsp) under the accession no. PRJCA007442 (Additional file 1: Table S2) [46]. After filtering with fastp [71], clean reads were mapped to the lettcue reference genome (Salinas_v8) using HiC-Pro (2.11.1) [83]. The aligned reads of both fragment mates were then paired in a single paired-end BAM file generated by HiC-Pro (2.11.1) [83]. Subsequently, dangling-end reads, same-fragment reads, self-circled reads, self-ligation reads, and other invalid Hi-C reads were discarded, and potential PCR duplicates were removed. Retained valid paired-end reads were used to generate raw Hi-C matrix by HiC-Pro (2.11.1) [83]. These matrices were normalized by the iterative correction and eigenvector decomposition (ICE) method of HiC-Pro (2.11.1) [83]. The normalized Hi-C matrices were used to identify intra-chromosomal interaction loops (q-value <0.05) by the “FitHiC” package (2.0.7) with default parameters [84].

### RNA-seq library construction and analysis

Total RNA was extracted from the same batch of plant tissues used for MethylC-seq and DNA-seq analysis. RNA-seq libraries were constructed as previously reported [82]. After DNase I treatment, aliquots of total RNA were individually fragmented. First-strand cDNA synthesis was performed using oligo(dT)-index primer, followed by second strand cDNA synthesis, end-repair, and adapter ligation. Subsequently, cDNA was amplified by 15 cycles of PCR reaction using Q5 HiFi HotStart DNA Polymerase (NEB). After purification, the 3′ RNA-seq libraries were sequenced on NovaSeq platform (Illumina) to generate 150 bp paired-end reads.

After filtering with fastp (v0.23.2) [71], clean reads were mapped to the lettuce reference genome (Salinas_v8) using Hisat2 (v2.2.1) [85]. After removing potential PCR duplicates using Picard-tools (version 2.0.1), uniquely mapped reads on each gene were normalized as reads per million mapped reads (RPM) to represent the expression level for each gene using featureCounts (v2.0.4) [86] with default parameters.

### 5-Azacytidine (5-AzaC) treatment

Seeds of the wild lettuce (*L. serriola*) were placed on sterile filter papers soaked in water with or without 5-AzaC (20 mg/L) and incubated in a climate-controlled chamber with a 16 h light/8 h dark cycle at 24 °C (day)/22°C (night). After 3 days after sowing (DAS), the old filter papers were replaced with new ones that soaked in water with or without 5-AzaC (20 mg/L). On 6 DAS, these wild lettuce seeds were transferred to soil and grown in a growth chamber under 16 h light/8 h dark at 24°C (day)/22 °C (night). The third pair of leaves at 30 DAS was collected at ZT4 (Zeitgeber time) for isolating genomic DNA and RNA. The mock- and 5-AzaC-treated genomic DNAs were used for constructing MethylC-seq libraries as described above, followed by sequencing on the NovaSeq platform (Illumina) to generate 150 bp paired-end reads. MethylC-seq data were analyzed as described above, and the CG methylation levels of Chr1: 59,076,400–59,077,800 were shown in Additional file 2: Fig. S13a.

### Functional validation of LsERF48

The full-length coding sequence of *LsERF48* was amplified with P1F and P1R (Additional file 1: Table S15) and cloned into the pCAMBIA1300-35S:GFP vector to generate *35S:LsERF48-GFP*. *Agrobacterium* culture (OD_600_ = 0.9) harboring *35S:LsERF48-GFP* or the empty *35S:GFP* vector were infiltrated into leaves of *N. benthamiana* at 30 days after planting. After 2 days post-inoculation (dpi), *N. benthamiana* with inoculated leaves were irrigated with water containing 150 mM NaCl (salt treatment) or 0 mM NaCl (mock treatment). After 4 dpi, ROS accumulation was analyzed using NBT (Nitro tetrazolium blue chloride) and DAB (diaminobenzidine) staining [87]. For DAB staining, salt- and mock-treated infiltrated *N. benthamiana* leaves were stained with DAB-HCl (1 mg/mL) solution overnight in darkness. Subsequently, the stained leaves were washed five times with sterile water, followed by fixation in 70% (v/v) ethanol for 10 h at room temperature. Decolorization was then performed in 96% ethanol at 40 °C to remove chlorophyll. For NBT staining, detached leaves were immersed in 100 mL of staining solution containing 0.05% (w/v) NBT, 10 mM sodium azide, 50 mM potassium phosphate, pH 6.4 for 6 h. After stopping the reaction with 95% ethanol, the samples were decolorized in 96% ethanol at 40 °C. The photos were taken under a light microscope. The IMAGEJ software (http://rsbweb.nih.gov/ij) was used to measure the relative intensities of DAB staining and NBT staining.

### qPCR analysis

Total RNA was extracted using the RNeasy Plant Mini Kit (Qiagen) and reverse transcribed with the HiScript III RT SuperMix (Vazyme) according to the manufacturer’s instructions. qPCR was performed using Taq Pro Universal SYBR Green Master Mix (Vazyme) on the CFX Opus 384 Real-Time PCR System (Bio-Rad). Relative gene expression levels were determined as previously described [88], with *LsActin* serving as the internal control. Primers used for qPCR analysis are listed in Additional file 1: Table S15.

### Supplementary Information

Supplementary Material 1. 

Supplementary Material 2. 

Supplementary Material 3. Review history.

## Data Availability

All high-throughput sequencing data including MethylC-seq, DNA-seq, RNA-seq, DAP-seq, and ATAC-seq data generated in this study were deposited into the Genome Sequence Archive (GSA) in BIG Data Center (https://bigd.big.ac.cn/gsa/index.jsp) under the accession number PRJCA017183  [89]. No other scripts and software were used other than those mentioned in the “Methods” section.
