# Supplementary material for: DNA methylation variations underlie lettuce domestication and divergence
Source: Genome Biol. 2024 Jun 17;25:158. doi: 10.1186/s13059-024-03310-x (PMC11184767; doi:10.1186/s13059-024-03310-x)
Supplement: Supplementary file 2 — Supplementary Material 2. [file 13059_2024_3310_MOESM2_ESM.pdf]

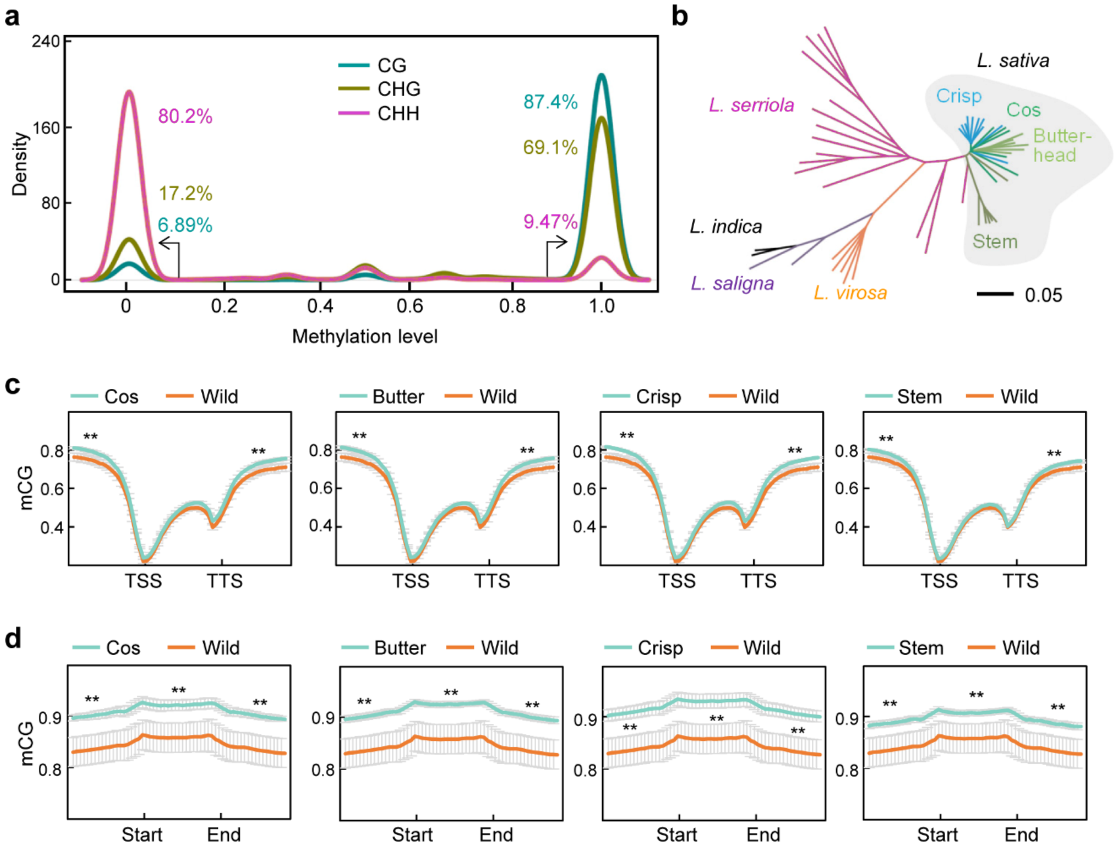

Fig. S1 Increased CG methylation levels during lettuce domestication. **a** Frequency of average methylation levels in different cytosine contexts for each cytosine. Bent arrows indicate the cutoff of methylation levels used for calculating the frequencies of average methylation levels. **b** A neighbor-joining phylogenetic tree of 52 *Lactuca* accessions based on SNPs. **c,d** Average CG methylation levels around genes (**c**) and TEs (**d**). Asterisks indicate significant differences (**$P < 0.01$, Wilcoxon signed-rank test) of CG methylation levels in cultivars as compared with the wild lettuce *L. serriola* (wild).

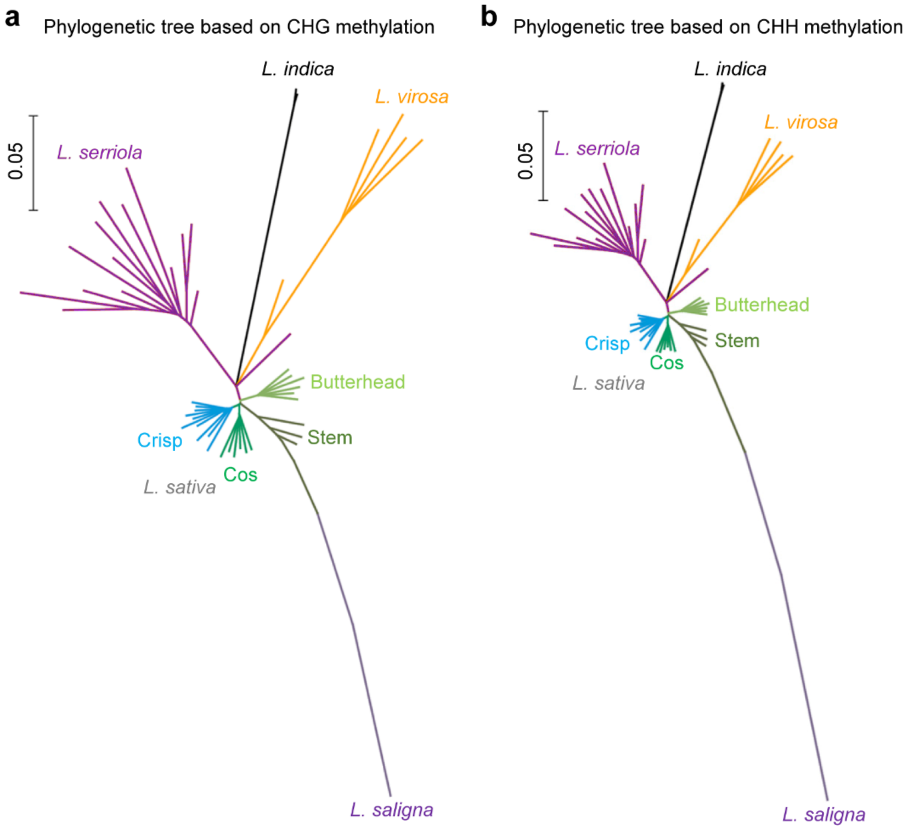

Fig. S2 Phylogenetic trees based on the methylation levels of all CHG and CHH loci. **a,b** A neighbor-joining phylogenetic tree of 52 *Lactuca* accessions based on the methylation levels of all CHG (**a**) and CHH (**b**) loci.

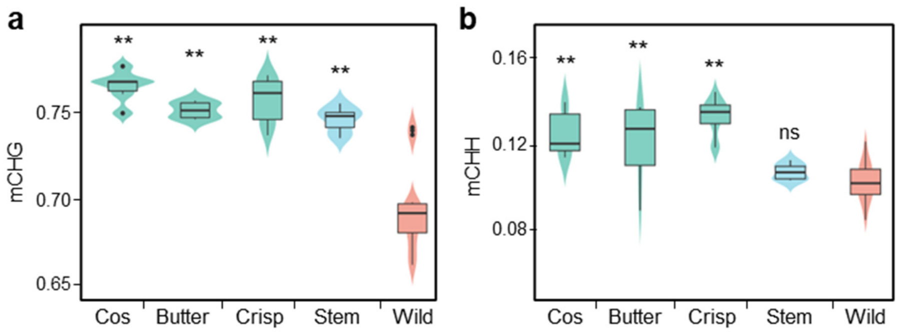

Fig. S3 Methylation levels of CHG and CHH during lettuce domestication. **a, b** Increased methylation levels of CHG (**a**) and CHH (**b**) in cultivated lettuces compared to the wild lettuce *L. serriola* (wild). Asterisks and ns indicate significant differences (**$P < 0.01$, two-tailed paired Student's t-test) and no statistical difference ($P \geqslant 0.01$, two-tailed paired Student's t-test) of methylation levels (mCHG and mCHH) in indicated lettuce types as compared with the wild lettuce *L. serriola*, respectively.

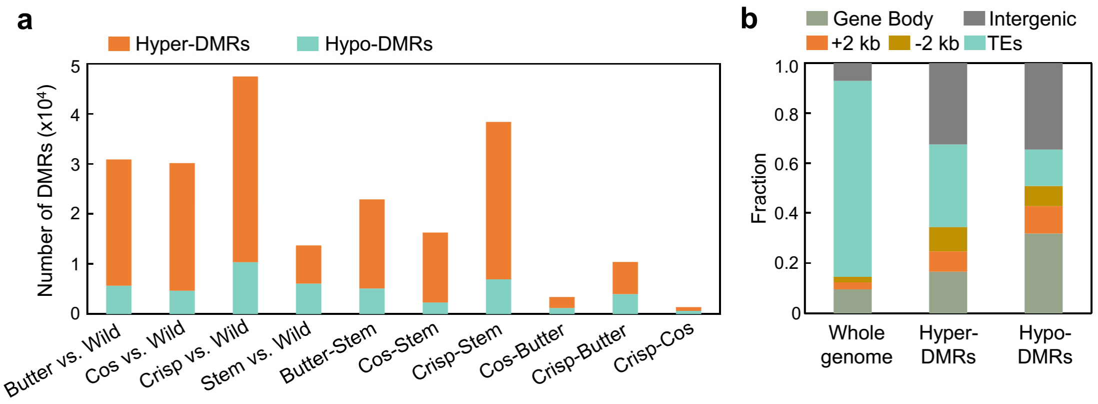

Fig. S4 Identification of DMRs in lettuce cultivars. **a** Number of DMRs in pairwise comparisons among the wild lettuce *L. serriola* (wild), butterhead (butter), cos, crisp, and stem lettuce. **b** Distribution of high-fidelity leafy-specific DMRs in different genomic regions divided into gene body, +2 kb flanking region (2 kb upstream of TSS), -2 kb flanking region (2 kb downstream of TTS), TEs, and other intergenic regions excluding TEs.

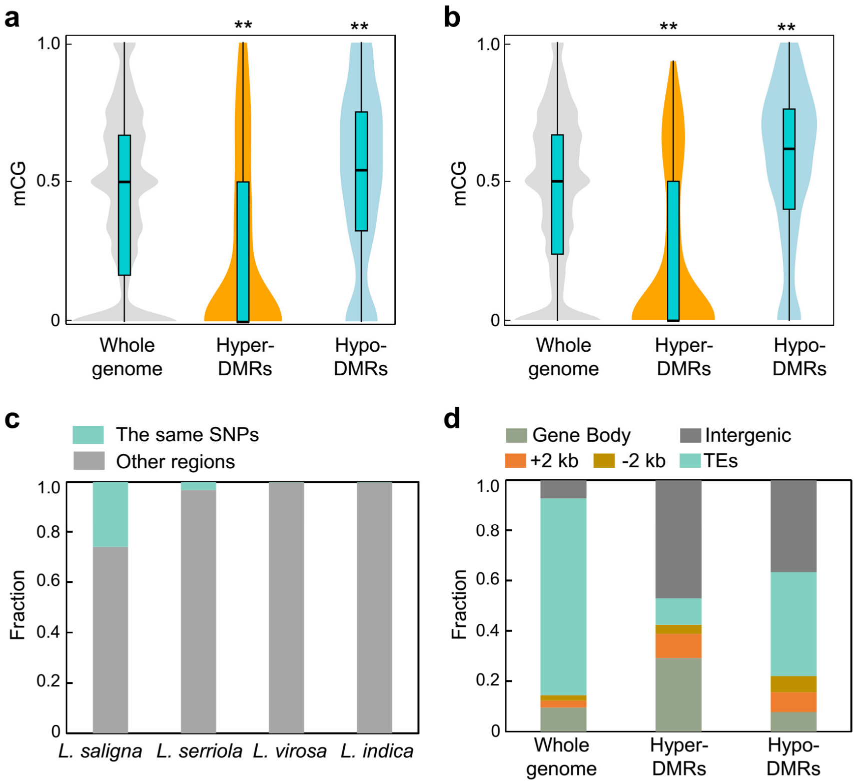

Fig. S5 Analysis of high-fidelity stem-specific DMRs. **a,b** Methylation levels of the high-fidelity stem-specific DMRs in the wild relatives *L. virosa* (**a**) and *L. indica* (**b**). Asterisks indicate significant differences (**$P < 0.01$, Wilcoxon signed-rank test) as compared to mCG levels in the whole genome. **c** Fraction of regions with the same SNPs in stem lettuce and a wild relative (either *L. saligna*, *L. serriola*, *L. virosa*, or *L. indica*) in the global windows for DMR identification. Blue bars indicate fraction of regions with the same SNPs between stem lettuce and one wild relative, but different from other cultivars (cos, butter, and crisp lettuce) and the other wild relatives. **d** Distribution of the high-fidelity stem-specific DMRs in different genomic regions divided into gene body, +2 kb flanking region (2 kb upstream of TSS), and -2 kb flanking region (2 kb downstream of TTS), TEs, and other intergenic regions excluding TEs.

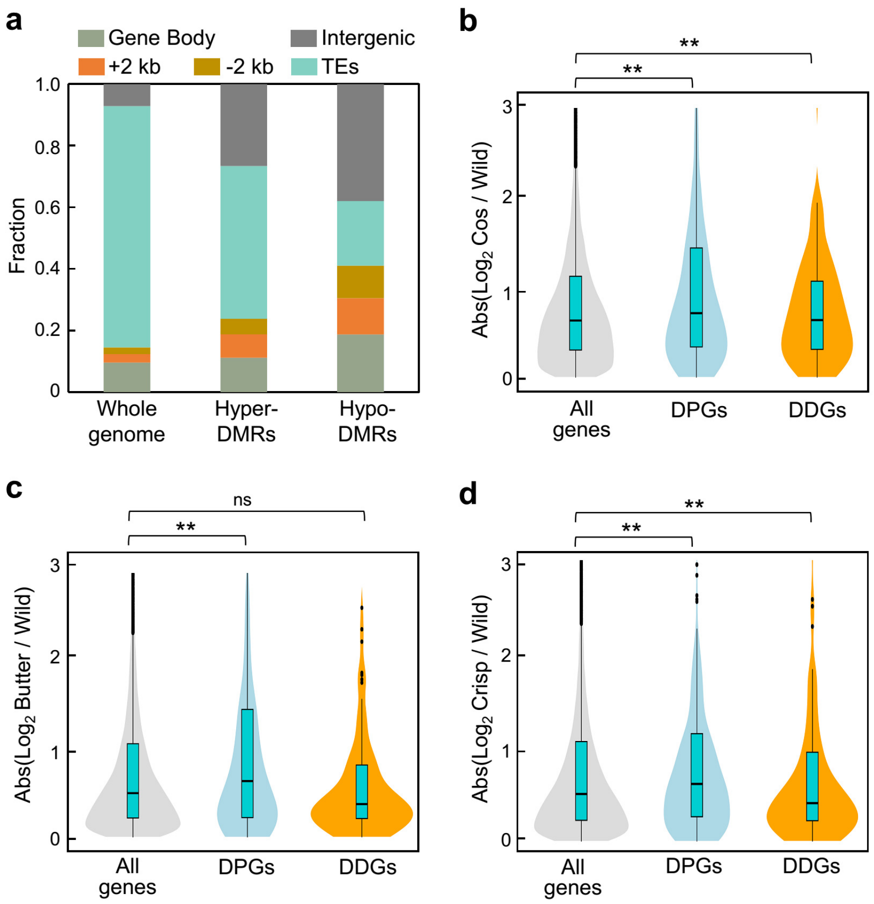

Fig. S6 Shared domestication-induced DMRs in lettuce cultivars influence gene expression. **a** Distribution of shared domestication-induced DMRs in different genomic regions divided into gene body, +2 kb flanking region (2 kb upstream of TSS), -2 kb flanking region (2 kb downstream of TTS), TEs, and other intergenic regions excluding TEs. **b-d** Absolute expression changes in cos (**b**), crisp (**c**), and butterhead (butter) lettuce (**d**) relative to the wild relative *L. serriola* (wild) in DMR-associated proximal genes (DPGs) and distal genes (DDGs) compared with all genes. Asterisks and ns indicate significance differences ($P < 0.01$, Wilcoxon signed-rank test) and no statistical differences ($P \geq 0.01$, Wilcoxon signed-rank test), respectively.

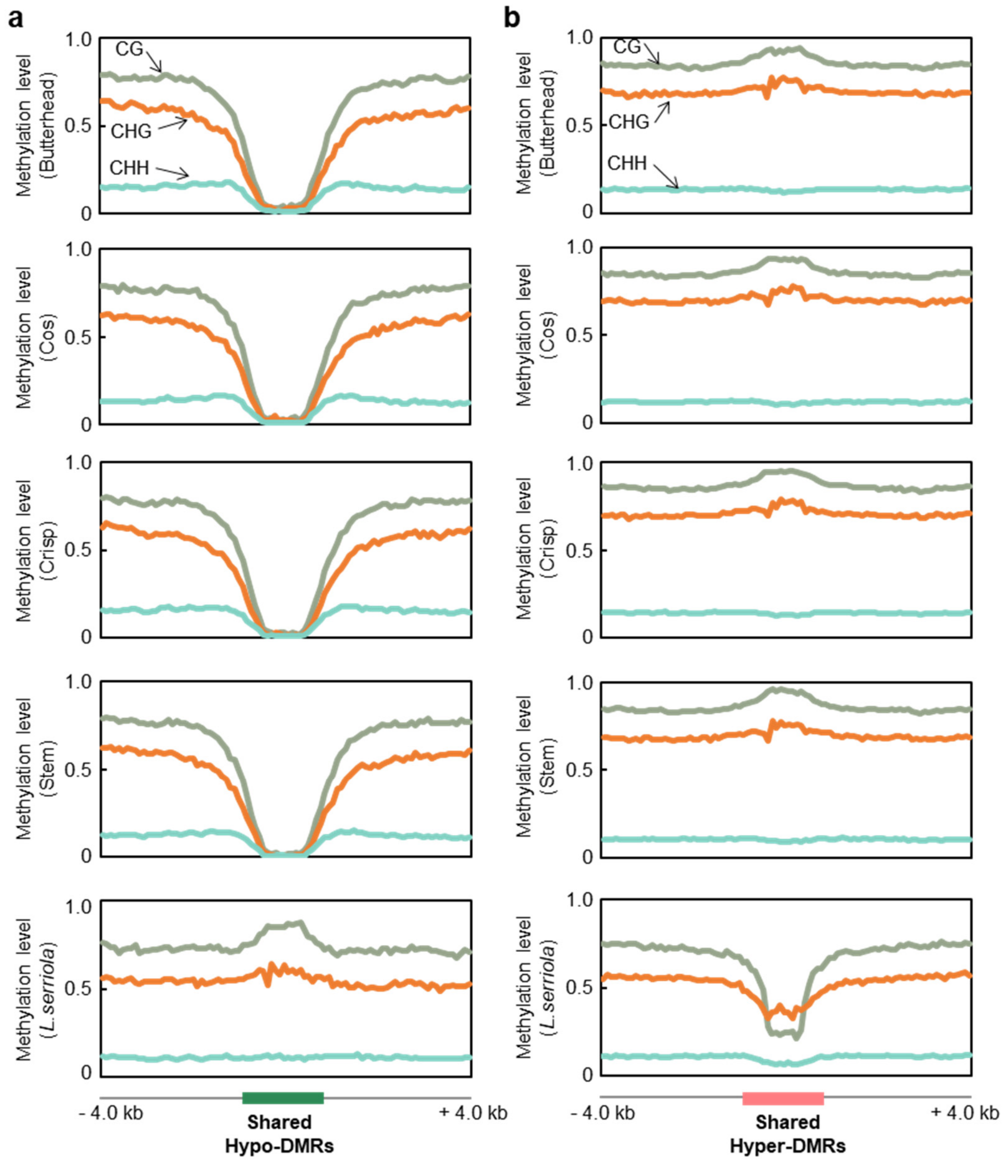

Fig. S7 Methylation levels of CG, CHG and CHH on the shared CG DMRs. **a, b** Methylation levels of CG, CHG and CHH on the shared hypo-DMRs (**a**) and shared hyper-DMRs (**b**).

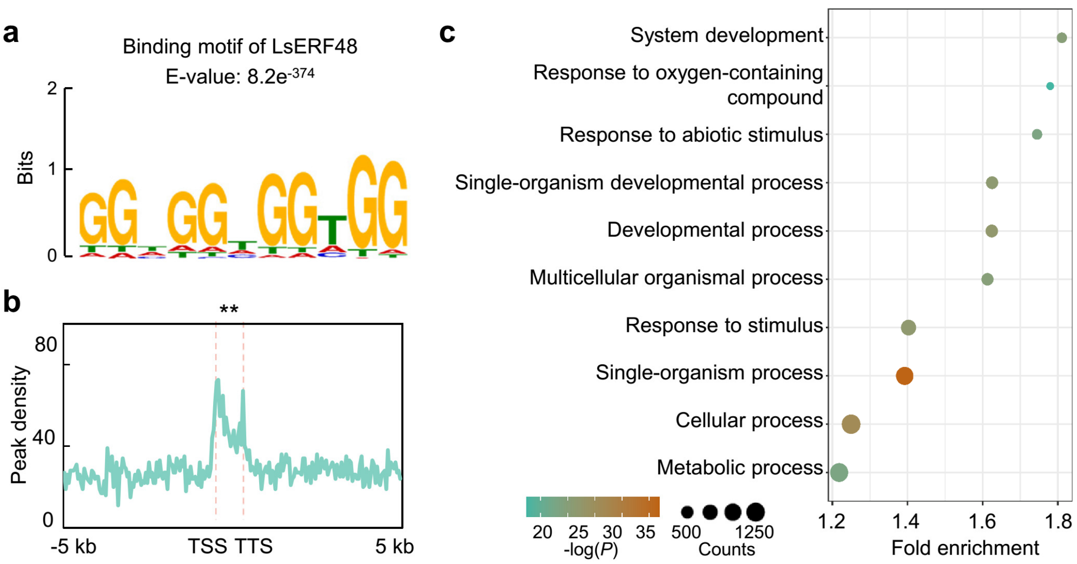

Fig. S8 DAP-seq analysis of LsERF48. **a** DNA motif enriched in LsERF48-bound peaks. **b** Density of LsERF48-bound peaks within 10 kb of genes. Asterisk indicates a significant difference (**$P < 0.01$, Wilcoxon signed-rank test) between gene regions and flanking regions. **c** GO enrichment analysis of LsERF48 target genes.

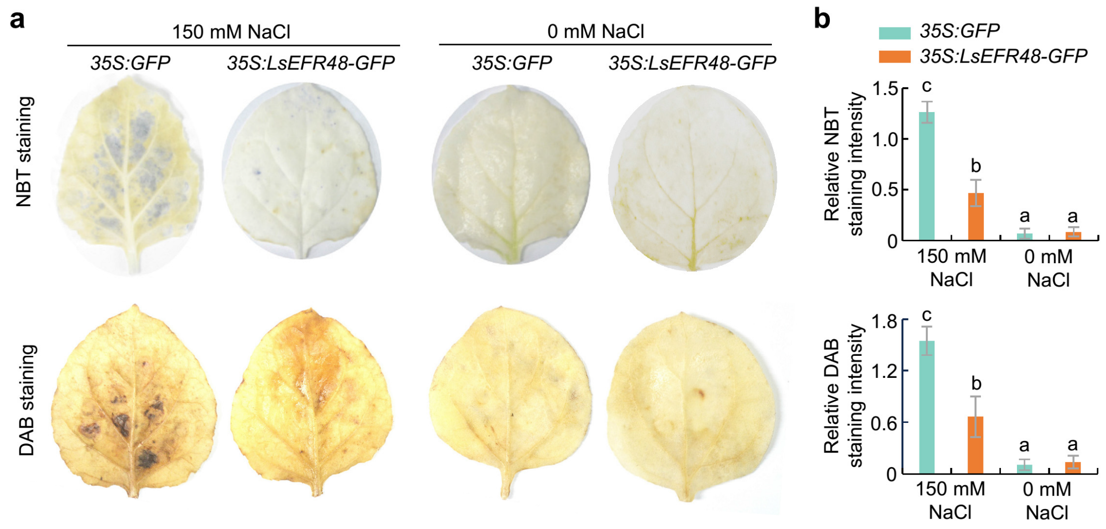

Fig. S9 Function of LsEFR48 related to ROS scavenging under salt stress. **a** In situ detection of ROS in *N. benthamiana* leaves under 150 mM or 0 mM NaCl treatment. **b** Quantification of relative NBT (upper panel) and DAB (lower panel) staining intensities as shown in (a) respectively. Different letters indicate significant differences ($P < 0.01$, two-tailed paired Student's t-test) in a pairwise comparison.

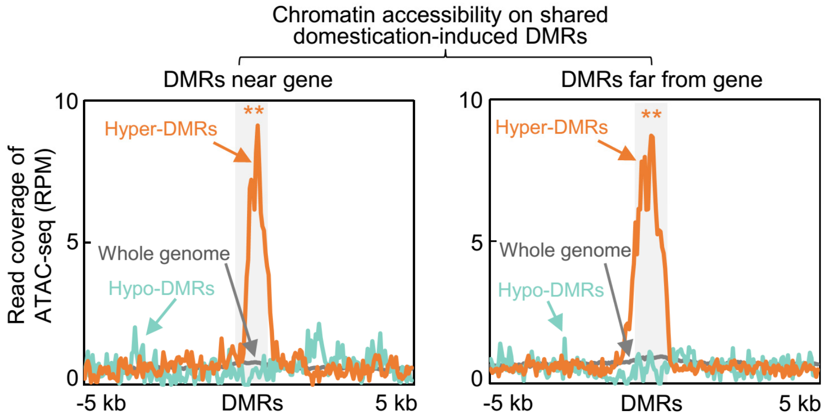

Fig. S10 Chromatin accessibility of the wild lettuce *L. serriola*. **a, b** Metaplots showing chromatin accessibility of wild lettuce *L. serriola* on the shared domestication-induced DMRs, located near the genes (**a**) and far from genes (**b**). Asterisks indicate significant differences (**$P < 0.01$, Wilcoxon signed-rank test) of the chromatin accessibility in DMRs compared with the whole genome.

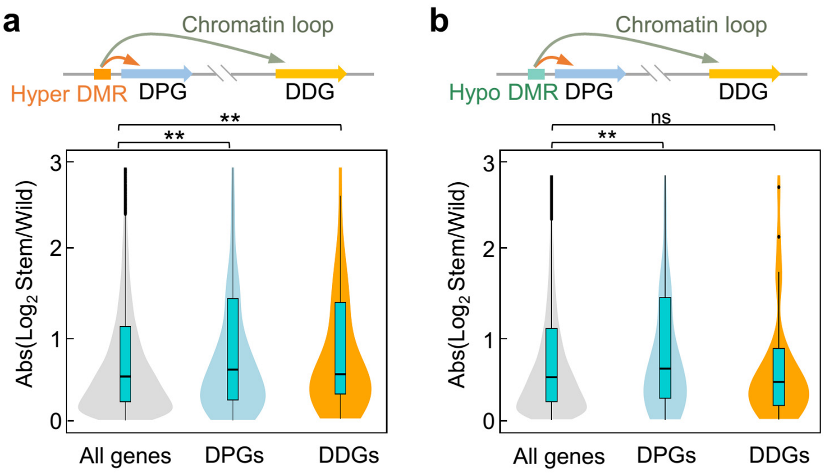

Fig. S11 Expression changes of genes related to shared hyper- or hypo-DMRs. **a, b** Absolute changes for gene expression between stem lettuce and the wild lettuce *L. serriola* in hyper- (**a**) and hypo- (**b**) DMR-associated proximal genes (DPGs) and distal genes (DDGs) compared with all genes. Asterisks indicate significant differences (**$P < 0.01$, Wilcoxon signed-rank test).

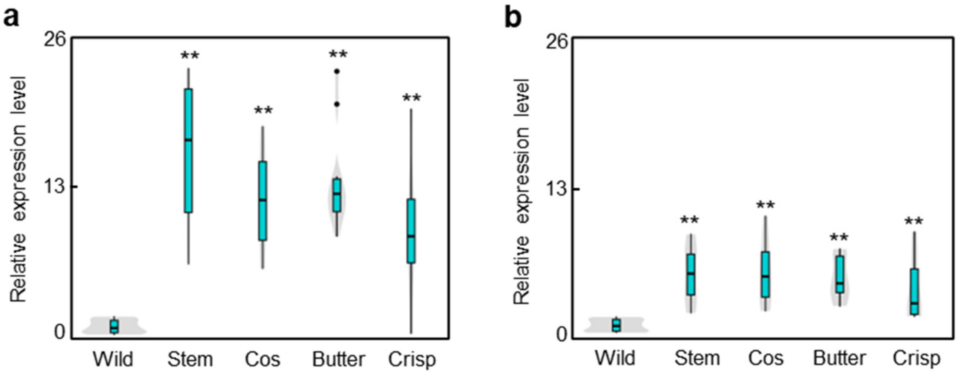

Fig. S12 Verification of gene expression changes by qPCR. **a**,**b** The relative expressions of *Lsat_1_v5_gn_1_50480* (**a**) and *Lsat_1_v5_gn_1_50600* (**b**) in cultivated lettuces (Stem, Cos, Butter, and Crisp) compared to the wild lettuce *L. serriola* (Wild). Double asterisks indicate significant difference (**$P < 0.01$, two-tailed paired Student's t-test).

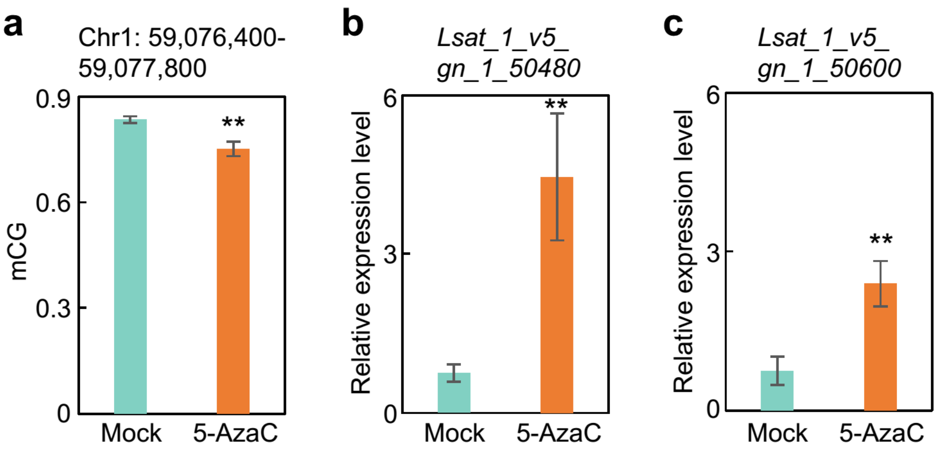

Fig. S13 5-Azacytidine (5-AzaC) treatment upregulates the expression of *Lsat_1_v5_gn_1_50480* and *Lsat_1_v5_gn_1_50600* in wild lettuce. **a** 5-AzaC treatment results in decreased DNA methylation level on the Chr1: 59,076,400 - 59,077,800 of wild lettuce. **b,c** 5-AzaC treatment leads to elevated expression levels of *Lsat_1_v5_gn_1_50480* (**b**) and *Lsat_1_v5_gn_1_50600* (**c**). Asterisks indicate significant difference (**$P < 0.01$, two-tailed paired Student's *t*-test).

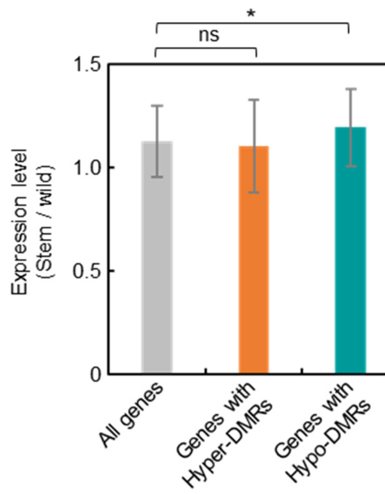

Fig. S14 Expression levels of genes related to shared DMRs between stem lettuce and the wild lettuce *L. serriola*. Asterisks and ns indicate significant difference (*$P < 0.05$, Wilcoxon signed-rank test) and no statistical difference ($P \geqslant 0.05$, Wilcoxon signed-rank test), respectively.
