# Supplementary material for: DNA methylation variations underlie lettuce domestication and divergence
Source: Genome Biol. 2024 Jun 17;25:158. doi: 10.1186/s13059-024-03310-x (PMC11184767; doi:10.1186/s13059-024-03310-x)
Supplement: Supplementary file 3 — Supplementary Material 3. Review history. [file 13059_2024_3310_MOESM3_ESM.docx]

Review History

**First round of review**

**Reviewer 1**

**Were you able to assess all statistics in the manuscript, including the appropriateness of statistical tests used?**

Yes, and I have assessed the statistics in my report.

**Comments to author:**

This study explored the epigenetic basis of lettuce domestication. They found lots of DMRs that show consistent higher or lower CG methylation levels in domesticated cultivars compared with wild progenitors. They also identified DMRs that are potentially associated with divergence among domesticated leafy and stem lettuces. Overall, this is a very interesting study and the authors did a good job in describing the results clearly. But there are some questions that need to be addressed.

1. I am worried that the sequencing depth of DNA methylation is not deep enough for DMR identification. Can you explain why you choose to sequence at this shallow depth? Have you ever done any quality control to show that DMRs can be robustly called at this sequencing depth?

2. It is interesting that L. saligna grouped differently on the phylogenetic tree built upon either the methylation data or the SNP data. And this study suggests a possible introgression of L. saligna genomic region into domesticated stem lettuce. I am curious, among the regions that are same between stem lettuce and L. saligna based on SNP data, are there any genes that are related to DNA methylation? The hypothesis is that if DNA methylation related genes were introgressed into stem lettuce from L. saligna, it may change the DNA methylation patterns of stem lettuce and makes it similar to L. saligna, which was observed in this study.

3. The authors identified shared Hyper- and Hypo- DMRs of the lettuce cultivars (Figure 5a). Among the genes that are associated with these DMRs, are there any genes that have been reported to be associated with lettuce domestication, e.g. evidence to be under selection.

4. Among the genes that are associated with DMRs between stem and leafy lettuce (Figure 4a), are there any genes that has been reported to be related to the divergence between these two types of lettuce? For example, are there any RNA-seq data to show the expression difference of these genes between stem and leafy lettuce? Are there any literatures that have reported the function of these genes? It would be very interesting to provide more evidence to show that DMRs are associated with domestication (question 3) or divergence (question 4).

5. L164, which DMRs were used? Are you using the Hyper- or/and hypo- DMRs?

6. L219-L221, I think this sentence doesn't describe what was shown in Figure 4c. Do you mean, "of the high-fidelity stem-specific DMRs, 41% and 48% overlapped with windows where stem lettuce shares same genotypes as L. saligna"?

7. L254, are there any public data available to show gene expression changes in erf48 mutant? Alternatively, the authors can use DAP-seq or similar method to confirm whether ERF48 can bind to these genes as suggested in the manuscript.

8. Figure 1b, which CG sites were used? Are you using all CG sites or CGs sites with methylation differences? Are you using individual CG sites or CG windows that have several CG sites? How do you quantify CG methylation? What would this plot look like if using CHG or CHH methylation?

9. Figure 6a, this plot is nice to show chromatin accessibility differences. Have you ever looked at the wild species? During domestication, how chromatin accessibility changed in these regions with DNA methylation changes?

10. Figure 6b, Are the cultivars that are used for RNA-seq the same as that used for DNA methylation? For hyper-DMRs, is there a tendency for down-regulation of gene expression, or vice versa?

**Reviewer 2**

**Were you able to assess all statistics in the manuscript, including the appropriateness of statistical tests used?**

Yes, and I have assessed the statistics in my report.

**Comments to author:**

Manuscript of "Evolution of DNA methylation during lettuce domestication and divergence" analyzed the DNA methylation variation during lettuce domestication and divergence based on 52 lettuce accessions. And authors found a significant increase of DNA methylation during lettuce domestication, but what effect does this increase have on trait during lettuce domestication? This research will be meaningful for understanding the lettuce domestication from an epigenetic perspective. However, it still have sevral problems in methods and results need to be revised.

1. Line 108, "cytosines at single nucleotide polymorphism (SNP) loci were removed for further analysis". Why authors removed these cytosines, it will lose many interesting cytosines with methylation information when you do this.

2. Line 111, it is understandable that CHH methylation is removed in further analysis, because the methylation level of CHH is too low. However, why CHG methylation is also removed in Manuscript when its methylation levels are not low. It should be pay more attention on this issue, and you can refer to these papers.

DOI: 10.1038/s41467-020-19333-4

DOI: 10.1007/s11427-022-2299-5

3. Fig 2, Can the author make reasonable explanations about the results of tree based on methylation. What are the reasons that L. saligna are clustered to the stem lettuce.

4. In fig 1d, the authors compare the diversity of mCG and "SNPs". How the SNPs set is generated. The diversity of SNPs in different genome background were different. Moreover, in line 108, the removal of cytosines has an impact on the genetic diversity of mCG, if the author has taken this into account. The authors are advised not to compare polymorphisms at a single site, but to compare them between regions, for example, DMR?

5. Line 136, the author reported an increase of CG methylation during lettue domestication. What's the changing pattern of CHG methylation?

6. Line157, the author's description is inconsistent with the results of the figure. In fig2d, most hyper-DMRs are enriched in the TEs interval. Moreover, the proportion of gene body was larger than the +2kb and -2kb intervals. However, the author said that it was preferentially enriched in intergenic regions and 5 'and 3' flanking regions. Please check your data and result.

7. Line 167, "lower correlation coefficients of 0.73 - 0.78 were found between stem lettuce and leafy types". 0.73-0.78 correlation should not be low. It seems unreasonable for the authors to draw conclusions about stem lettuce and leafy types of lettuce based on the correlation of CG methylation. Moreover, the results of tree and PCA in fig. 1b and 1c showed that stem is clustered in the leafy type. The DNA methylation separation of stem lettuce and leafy types lettuce may be smaller.

8. Line 172, it seems that there are also many DMRs shared both stem and leafy (cos, butterhead, and crisp) types, such as hyper-DMRs (fig 3b), 997+2826+598+619 shared in stem and leafy types. Moreover, in line 174 and fig3 d, most stem and leafy types specific DMRs exhibited same changing patterns compared to wild lettuce. Therefore, it may not be appropriate to propose independent domestication of stem and leafy types lettuce from DNA methylation perspective.

9. Line 202-211, it is difficult to understand the point of the author analyzing these?

10. In fact, it is interesting to reveal the effect of DNA methylation domestication on the formation of lettuce domestication traits, but the author only did GO enrichment analysis without in-depth analysis of genes, it deserve to explore deeply.

11. Line 279, "DNA methylation changes significantly induced expression changes of both DPGs and DDGs in stem lettuce". Whether the hyper-DMRs or hypo-DMRs induced gene expression? It is suggested that the author divide into two categories (hyper-DMRs or hypo-DMRs) to see.

12. Line 282, why the authors selected Lsat_1_v5_gn_1_50480 and Lsat_1_v5_gn_1_50600 as case genes? What roles do Lsat_1_v5_gn_1_50480 and Lsat_1_v5_gn_1_50600 play during lettuce domestication? It should verify the expression regulation of DNA methylation for candidate genes using 5-Aza or LUC/REN, and the function of candidate genes.

**Authors Response**

**Point-by-point responses to the reviewers’ comments:**

Reviewer #1

This study explored the epigenetic basis of lettuce domestication. They found lots of DMRs that show consistent higher or lower CG methylation levels in domesticated cultivars compared with wild progenitors. They also identified DMRs that are potentially associated with divergence among domesticated leafy and stem lettuces. Overall, this is a very interesting study and the authors did a good job in describing the results clearly. But there are some questions that need to be addressed.

>Reviewer:

1. I am worried that the sequencing depth of DNA methylation is not deep enough for DMR identification. Can you explain why you choose to sequence at this shallow depth? Have you ever done any quality control to show that DMRs can be robustly called at this sequencing depth?

*>Author:*

*A previous report on maize methylomes examined the influence of different sequencing depths on assessing DNA methylation and showed that a sequencing depth as low as ~2x genome coverage could effectively detect DNA methylation profiles (Li et al., 2014). Given that lettuce has a large genome size of approximately 2.5 Gb, which is similar to maize (~2.3 Gb), we used a higher sequencing depth, and the current sequencing depth of ~6x genome coverage on average should be sufficient for DNA methylation analysis.*

*Relevant reference*

*Li Q, Eichten SR, Hermanson PJ, Zaunbrecher VM, Song J, Wendt J, Rosenbaum H, Madzima TF, Sloan AE, Huang J, et al: Genetic perturbation of the maize methylome. Plant Cell 2014, 26:4602-4616.*

>Reviewer:

2. It is interesting that L. saligna grouped differently on the phylogenetic tree built upon either the methylation data or the SNP data. And this study suggests a possible introgression of L. saligna genomic region into domesticated stem lettuce. I am curious, among the regions that are same between stem lettuce and L. saligna based on SNP data, are there any genes that are related to DNA methylation? The hypothesis is that if DNA methylation related genes were introgressed into stem lettuce from L. saligna, it may change the DNA methylation patterns of stem lettuce and makes it similar to L. saligna, which was observed in this study.

*>Author:*

*As suggested, we have checked genes located in the regions related to the introgression of L. saligna and found that these regions do not contain known genes relevant to regulation of DNA methylation, implying the possible existence of other epigenetic evolution mechanism(s) in stem lettuce in relation to L. saligna. Among the genes we analyzed are those encoding methylating and demethylating enzymes, as well as regulatory factors such as homologs of MET1 (Lsat_1_v5_gn_7_75121), CMT2 (Lsat_1_v5_gn_3_37841, Lsat_1_v5_gn_5_63821), CMT3 (Lsat_1_v5_gn_4_68381, Lsat_1_v5_gn_6_36561), DDM1 (Lsat_1_v5_gn_2_76200, Lsat_1_v5_gn_3_18100, Lsat_1_v5_gn_5_163080) and demeter-like genes (Lsat_1_v5_gn_6_57640, Lsat_1_v5_gn_6_95000, Lsat_1_v5_gn_9_79861, Lsat_1_v5_gn_2_21401, Lsat_1_v5_gn_4_121940).*

>Reviewer:

3. The authors identified shared Hyper- and Hypo- DMRs of the lettuce cultivars (Figure 5a). Among the genes that are associated with these DMRs, are there any genes that have been reported to be associated with lettuce domestication, e.g. evidence to be under selection.

*>Author:*

*We thank the reviewer for this suggestion. The study of lettuce domestication is still in its infancy. Although abundant genetic variations during lettuce domestication have been profiled by a large-scale whole genome resequencing study (Wei et al., 2021), there are few examples of genes that have been shown to be directly linked to lettuce domestication. Characterized lettuce genes that are possibly involved in lettuce domestication include LsAP2 regulating seed shape (Luo et al., 2021), LsKN1 controlling leafy head development (Yu et al., 2020), and LsNRL4 regulating photosynthesis and leaf angles (An et al., 2022). Unfortunately, these genes do not contain shared DMRs.*

*While the limited progress in lettuce domestication has hindered our further analysis of the methylation status of domestication traits-associated genes, we have identified hundreds of genes that are associated with these domestication-induced DMRs, which will serve as valuable resources to facilitate future functional genomic studies in investigating domestication genes in lettuce.*

*Relevant references*

*An G, Qi Y, Zhang W, Gao H, Qian J, Larkin RM, Chen J, Kuang H: LsNRL4 enhances photosynthesis and decreases leaf angles in lettuce. Plant Biotechnol. J. 2022, 20:1956-1967.*

*Luo C, Wang S, Ning K, Chen Z, Wang Y, Yang J, Qi M, Wang Q: The APETALA2 transcription factor LsAP2 regulates seed shape in lettuce. J. Exp. Bot. 2021, 72:2463-2476.*

*Wei T, van Treuren R, Liu X, Zhang Z, Chen J, Liu Y, et al: Whole-genome resequencing of 445 Lactuca accessions reveals the domestication history of 24 cultivated lettuce. Nat. Genet. 2021, 53:752-760.*

*Yu C, Yan C, Liu Y, Liu Y, Jia Y, Lavelle D, An G, Zhang W, Zhang L, Han R, et al: Upregulation of a KN1 homolog by transposon insertion promotes leafy head development in lettuce. Proc. Natl. Acad. Sci. USA 2020, 117:33668-33678.*

>Reviewer:

4. Among the genes that are associated with DMRs between stem and leafy lettuce (Figure 4a), are there any genes that has been reported to be related to the divergence between these two types of lettuce? For example, are there any RNA-seq data to show the expression difference of these genes between stem and leafy lettuce? Are there any literatures that have reported the function of these genes? It would be very interesting to provide more evidence to show that DMRs are associated with domestication (question 3) or divergence (question 4).

*>Author:*

*Similar to the limited progress in lettuce domestication as mentioned above, there is currently no report of genes related to the divergence of leafy and stem lettuce. As suggested by this reviewer, we analyzed the expression levels of the genes related to the stem lettuce-specific DMRs in different types of cultivated lettuce and the wild lettuce L. serriola (Additional file 1: Table S13). This dataset, together with the differential DNA methylation data, serves as valuable resources for further functional genomic research on lettuce divergence.*

*Population epigenetic studies suggest roles of epigenetics evolution in the domestication processes of several crops including maize, soybean, cotton, and rice (Cao et al., 2023; Shen et al., 2018; Song et al., 2017; Xu et al., 2020; Xu et al., 2019). In crops with well-characterized functional genomics, such as rice, it is possible to identify traits-associated DMRs (Cao et al., 2023). Unfortunately, research on lettuce functional genomics lags behind, impeding the identification of traits-associated DMRs in lettuce domestication and divergence. Nevertheless, our study provides a valuable map of DNA methylation across diverse accessions, enhancing our understanding of lettuce domestication and divergence. We believe that the thousands of genes we have identified as associated with lettuce domestication and divergence will serve as valuable resources to facilitate future functional genomic studies to investigate traits-associated genes contributing to lettuce domestication and divergence.*

*Relevant reference*

*Cao S, Chen K, Lu K, Chen S, Zhang X, Shen C, et al: Asymmetric variation in DNA methylation during domestication and de-domestication of rice. Plant Cell. 2023.*

*Shen Y, Zhang J, Liu Y, Liu S, Liu Z, Duan Z, et al: DNA methylation footprints during soybean domestication and improvement. Genome Biol. 2018, 19:128.*

*Song Q, Zhang T, Stelly DM, Chen ZJ: Epigenomic and functional analyses reveal roles of epialleles in the loss of photoperiod sensitivity during domestication of allotetraploid cottons. Genome Biol. 2017, 18:99.*

*Xu G, Lyu J, Li Q, Liu H, Wang D, Zhang M, et al: Evolutionary and functional genomics of DNA methylation in maize domestication and improvement. Nat. Commun. 2020, 11:5539.*

*Xu J, Chen G, Hermanson PJ, Xu Q, Sun C, Chen W, et al: Population-level analysis reveals the widespread occurrence and phenotypic consequence of DNA methylation variation not tagged by genetic variation in maize. Genome Biol. 2019, 20:243.*

>Reviewer:

5. L164, which DMRs were used? Are you using the Hyper- or/and hypo- DMRs?

*>Author:*

*We used all DMRs including both hyper- and hypo-DMRs. We have clearly indicated it in the revised manuscript.*

>Reviewer:

6. L219-L221, I think this sentence doesn't describe what was shown in Figure 4c. Do you mean, "of the high-fidelity stem-specific DMRs, 41% and 48% overlapped with windows where stem lettuce shares same genotypes as L. saligna"?

*>Author:*

*We agree with this reviewer and have changed this sentence to “41% and 48% of high-fidelity stem-specific hyper-DMRs and hypo-DMRs, respectively, overlapped with the identified windows where stem lettuce shares the same genotypes as L. saligna.” in the revised manuscript.*

>Reviewer:

7. L254, are there any public data available to show gene expression changes in erf48 mutant? Alternatively, the authors can use DAP-seq or similar method to confirm whether ERF48 can bind to these genes as suggested in the manuscript.

*>Author:*

*We thank the reviewer for this suggestion. Since there is no publicly available data on gene expression changes in the erf48 mutant, we have performed a DAP-seq analysis to profile target genes of LsERF48 (Lsat_1_v5_gn_8_13141) in the revised manuscript (Additional file 1: new Table S12; Additional file 2: new Fig. S8). A GGWGGTGGTGG motif was found to be enriched in the binding sites of LsERF48 (Additional file 2: new Fig. S8a), similar to the motifs found in AtERF48 binding sites and shared DMRs (Fig. 5d). Consistent with the motif distribution of shared DMRs (Fig. 5e), LsERF48 binding sites are more prevalent in gene regions (Additional file 2: new Fig. S8b). Interestingly, LsERF48 target genes are also enriched in stress response (Additional file 2: new Fig. S8c). Remarkably, 38 out of 671 genes associated with shared DMRs are also LsERF48 targets (Additional file 1: Table S12). We have included the relevant figure and descriptions in the revised manuscript.*

>Reviewer:

8. Figure 1b, which CG sites were used? Are you using all CG sites or CGs sites with methylation differences? Are you using individual CG sites or CG windows that have several CG sites? How do you quantify CG methylation? What would this plot look like if using CHG or CHH methylation?

*>Author:*

*All individual CGs sites were used for constructing the phylogenetic tree shown in Fig. 1b. We have included this information in the revised sentence. The CG methylations levels were analyzed using the mixtools (Benaglia et al., 2009) under “normalmixEM”. The details have been included in the subsection of “Single cytosine methylation calling and population parameter (Pi, π)” in the “Methods” section. Furthermore, as suggested by this reviewer, we have constructed phylogenetic trees based on methylation levels of all CHG sites or CHH sites (Additional file 2: new Fig. S2), which are similar to that based on CG sites (Fig. 1b).*

*Relevant reference*

*Benaglia T, Chauveau D, Hunter DR, Young DS: mixtools: An R package for analyzing finite mixture models. J. Stat. Softw. 2009, 32:1-29.*

>Reviewer:

9. Figure 6a, this plot is nice to show chromatin accessibility differences. Have you ever looked at the wild species? During domestication, how chromatin accessibility changed in these regions with DNA methylation changes?

*>Author:*

*We thank the reviewer for raising this interesting question. In the revised manuscript, we have performed ATAC-seq on the wild lettuce (L. serriola). Notably, we observed high levels of chromatin accessibility in wild lettuce in shared hyper-DMRs near genes and far away from genes (Additional file 2: new Fig. S9), where wild lettuce possessed low DNA methylation levels (Fig. 5a). This observation, together with the relatively low chromatin accessibility in shared hyper-DMRs in stem lettuce that possessed high DNA methylations in these regions (Fig. 6a), indicate that domestication is associated with an increase in DNA methylation alongside a decrease in chromatin accessibility in stem lettuce compared to wild lettuce.*

>Reviewer:

10. Figure 6b, Are the cultivars that are used for RNA-seq the same as that used for DNA methylation? For hyper-DMRs, is there a tendency for down-regulation of gene expression, or vice versa?

*>Author:*

*The RNA-seq data were downloaded from the published study (Zhang et al, 2017), which includes a large number of lettuce accessions (28 butterhead, 19 crisp, 24 stem, 31 cos, and 24 L. serriola accessions). While the accessions used are not exactly the same, they allow us to assess the effect of DNA methylation from an evolutionary perspective.*

*We also analyzed expression levels of genes with hyper-DMRs and hypo-DMRs as suggested by this reviewer. We found that genes with hypo-DMRs showed significantly higher expression, but genes with hyper-DMRs display slightly lower expression (insignificant) in stem lettuce compared to wild lettuce (L. serriola) (Additional file: new Fig. S12).*

*Relevant reference*

*Zhang L, Su W, Tao R, Zhang W, Chen J, Wu P, Yan C, Jia Y, Larkin RM, Lavelle D, et al: RNA sequencing provides insights into the evolution of lettuce and the regulation of flavonoid biosynthesis. Nat. Commun. 2017, 8:2264.*

Reviewer #2

Manuscript of "Evolution of DNA methylation during lettuce domestication and divergence" analyzed the DNA methylation variation during lettuce domestication and divergence based on 52 lettuce accessions. And authors found a significant increase of DNA methylation during lettuce domestication, but what effect does this increase have on trait during lettuce domestication? This research will be meaningful for understanding the lettuce domestication from an epigenetic perspective. However, it still have sevral problems in methods and results need to be revised.

>Reviewer:

1. Line 108, "cytosines at single nucleotide polymorphism (SNP) loci were removed for further analysis". Why authors removed these cytosines, it will lose many interesting cytosines with methylation information when you do this.

*>Author:*

*Bisulfite treatment converts unmethylated cytosine (C) into uracil (U), which is subsequently converted to thymine (T) during PCR amplification in library preparation, while methylated cytosine remains unchanged. The DNA methylation level is determined by the ratio of mapped cytosine (C) to the sum of cytosine (C) plus bisulfite-converted thymine (T). SNPs at cytosines especially C to T could cause spurious methylation calls, and were thus excluded. This practice is common in generating DNA methylomes via whole-genome bisulfite sequencing (e.g. Jeong et al., 2021; Song et al., 2017). We also analyzed the SNP loci and found that cytosines at the SNP loci account for only 0.65% of the total cytosines, indicating that excluding cytosines at SNPs could have negligible impact on further analyses. Additionally, the methylation information of these excluded cytosines could also be represented by local DMRs containing multi-cytosines. We have revised this sentence to explain why cytosines at SNP loci were removed in the revised manuscript.*

*Relevant references*

*Jeong H, Mendizabal I, Berto S, Chatterjee P, Layman T, Usui N, Toriumi K, Douglas C, Singh D, Huh I, et al: Evolution of DNA methylation in the human brain. Nat. Commun. 2021, 12:2021.*

*Song Q, Zhang T, Stelly DM, Chen ZJ: Epigenomic and functional analyses reveal roles of epialleles in the loss of photoperiod sensitivity during domestication of allotetraploid cottons. Genome Biology 2017, 18:99.*

>Reviewer:

2. Line 111, it is understandable that CHH methylation is removed in further analysis, because the methylation level of CHH is too low. However, why CHG methylation is also removed in Manuscript when its methylation levels are not low. It should be pay more attention on this issue, and you can refer to these papers. DOI: 10.1038/s41467-020-19333-4；DOI: 10.1007/s11427-022-2299-5

*>Author:*

*We thank the reviewer for this suggestion. In the revised manuscript, we have further analyzed the CHG and CHH methylation. First, we used CHG and CHH methylation levels to construct phylogenetic trees (Additional file 2: new Fig. S2), both of which are similar to that based on CG methylation levels (Fig. 1b). Second, we calculated the average methylation levels of all CHG and CHH sites, and found globally increased CHG and CHH methylation levels during lettuce domestication (Additional file 2: new Fig. S3). Third, we compared CG, CHG, and CHH methylation levels in the CG-DMRs in cultivated lettuce and wild lettuce, and found consistent changes of CHG and CHH methylation levels with CG methylation between cultivated lettuce and wild lettuce (Additional file 2: new Fig. S7). Notably, shared hypo-DMRs with very low CG methylation levels also contain low levels of CHG and CHH methylation in cultivated lettuce (Additional file 2: new Fig. S7a). Collectively, these results indicate conserved changes of DNA methylation on different cytosine contexts during lettuce domestication. All these relevant figures and descriptions have been included in the revised manuscript.*

>Reviewer:

3. Fig 2, Can the author make reasonable explanations about the results of tree based on methylation. What are the reasons that L. saligna are clustered to the stem lettuce.

*>Author:*

*L. saligna and stem lettuce share some common DNA methylation features. When analyzing the DNA methylation levels of high-fidelity stem-specific DMRs in L. saligna, L. virosa and L. indica, we found that only L. saligna showed similar methylation changes to those in stem lettuce (Fig. 4b), while opposite changes were observed in two other wild relatives, L. virosa and L. indica (Additional file 2: Fig. S5a,b). These observations indicate the likely existence of a unique epigenetic evolution mechanism in stem lettuce in relation to L. saligna, which may result in the clustering of L. saligna with stem lettuce in the phylogenetic tree based on methylation levels (Fig. 1b). This information has been included in the manuscript.*

>Reviewer:

4. In fig 1d, the authors compare the diversity of mCG and "SNPs". How the SNPs set is generated. The diversity of SNPs in different genome background were different. Moreover, in line 108, the removal of cytosines has an impact on the genetic diversity of mCG, if the author has taken this into account. The authors are advised not to compare polymorphisms at a single site, but to compare them between regions, for example, DMR?

*>Author:*

*The whole genome-sequencing data of these accessions for SNP calling were generated in parallel with the whole-genome bisulfite sequencing data for CG methylation calling in this study. As mentioned* above, *cytosines at SNPs were excluded to avoid spurious methylation calls. Moreover, cytosines at the SNP loci account for only 0.65% of the total cytosines and were excluded from our analysis, which could have negligible effect on the diversity based on CG methylation variations shown in Fig. 1d.*

>Reviewer:

5. Line 136, the author reported an increase of CG methylation during lettue domestication. What's the changing pattern of CHG methylation?

*>Author:*

*As mentioned above, we have further analyzed CHG methylation and found an increased CHG methylation during lettuce domestication (Additional file 2: new Fig. S3a).*

>Reviewer:

6. Line157, the author's description is inconsistent with the results of the figure. In fig2d, most hyper-DMRs are enriched in the TEs interval. Moreover, the proportion of gene body was larger than the +2kb and -2kb intervals. However, the author said that it was preferentially enriched in intergenic regions and 5 'and 3' flanking regions. Please check your data and result.

*>Author:*

*One clarification is that we compared the proportions of DMRs in different genomic regions relative to their distributions in the whole genome (shown by the uppermost bar in Fig. 2d). For example, although the proportion of TEs in hyper-DMRs is higher than others genome features, it is less enriched as compared to the proportion of TE in the whole genome.*

>Reviewer:

7. Line 167, "lower correlation coefficients of 0.73 - 0.78 were found between stem lettuce and leafy types". 0.73-0.78 correlation should not be low. It seems unreasonable for the authors to draw conclusions about stem lettuce and leafy types of lettuce based on the correlation of CG methylation. Moreover, the results of tree and PCA in fig. 1b and 1c showed that stem is clustered in the leafy type. The DNA methylation separation of stem lettuce and leafy types lettuce may be smaller.

*>Author:*

*We agree with this reviewer that 0.73-0.78 correlation is not low. However, when comparing these correlation coefficients of 0.73-0.78 with those among leafy type lettuce (0.85-0.94), they are lower. We have revised this sentence to make this comparison clear in the revised manuscript. Moreover, although stem lettuce is clustered with the leafy type lettuce in the phylogenetic tree, stem lettuce is separated from the leafy types in the PCA plot based on CG methylation levels (enlarged plot in Fig. 1c).*

>Reviewer:

8. Line 172, it seems that there are also many DMRs shared both stem and leafy (cos, butterhead, and crisp) types, such as hyper-DMRs (fig 3b), 997+2826+598+619 shared in stem and leafy types. Moreover, in line 174 and fig3 d, most stem and leafy types specific DMRs exhibited same changing patterns compared to wild lettuce. Therefore, it may not be appropriate to propose independent domestication of stem and leafy types lettuce from DNA methylation perspective.

*>Author:*

*It is not surprising that stem and leafy lettuce possess many shared DMRs, since they are believed to be domesticated from a single wild ancestor, L. serriola, around 4,000 BC (Wei et al., 2021), which is also supported by our phylogenetic tree based on DNA methylation levels (Fig. 1b). However, it has been hypothesized that stem and leafy type lettuces evolved independently after the initial domestication event (Zhang et al., 2017). While they have a lot of shared DMRs, more than 5,000 DMRs are specific to stem lettuce (Fig. 4a), and most of these DMRs showed different changing patterns between stem and leafy lettuce. Our analysis of these stem- specific DMRs has revealed their unique features (Fig. 4; Additional file 2: Fig. S5). In addition, to avoid confusion, we emphasis the independent evolution or divergence rather than domestication of stem and leafy types in the revised manuscript. Thus, we hope the reviewer agree that our analysis of the type-specific DMRs support the idea of the independent evolution of stem lettuce after the initial domestication from the perspective of DNA methylation.*

*Relative references*

*Wei T, van Treuren R, Liu X, Zhang Z, Chen J, Liu Y, Dong S, Sun P, Yang T, Lan T, et al: Whole-genome resequencing of 445 Lactuca accessions reveals the domestication history of cultivated lettuce. Nat. Genet. 2021, 53:752-760.*

*Zhang L, Su W, Tao R, Zhang W, Chen J, Wu P, Yan C, Jia Y, Larkin RM, Lavelle D, et al: RNA sequencing provides insights into the evolution of lettuce and the regulation of flavonoid biosynthesis. Nat. Commun. 2017, 8:2264.*

>Reviewer:

9. Line 202-211, it is difficult to understand the point of the author analyzing these?

*>Author:*

*Although stem lettuce and L. saligna show distinct phenotypes (Fig. 1a) and huge nucleotide diversity as evident from the phylogenetic tree based on SNPs (Additional file: Fig. S1b), L. saligna were classified to the stem lettuce in the phylogenetic tree based on CG methylation levels (Fig. 1b). To understand this observation, we analyzed the methylation levels of high-fidelity stem-specific DMRs in L. saligna, L. virosa, and L. indica. We observed that stem lettuce shared similar features of DNA methylation with L. saligna, but not with L. virosa and L. indica (Fig. 4b; Additional file 2: Fig. S5a,b). These results indicate a possible existence of a unique epigenetic evolution mechanism in stem lettuce in relation to L. saligna. We have revised the text of lines 202-211 (line numbers in the original manuscript) to make it clearer.*

>Reviewer:

10. In fact, it is interesting to reveal the effect of DNA methylation domestication on the formation of lettuce domestication traits, but the author only did GO enrichment analysis without in-depth analysis of genes, it deserve to explore deeply.

*>Author:*

*We certainly agree with the reviewer that it is interesting to explore the effect of DNA methylation on the formation of lettuce domestication traits. However, the study of lettuce domestication is still in its infancy. Although abundant genetic variations during lettuce domestication have been profiled by a large-scale whole genome resequencing study (Wei et al., 2021), there are few examples of genes that have been shown to be directly linked to lettuce domestication. Characterized lettuce genes that are possibly involved in lettuce domestication include LsAP2 regulating seed shape (Luo et al., 2021), LsKN1 controlling leafy head development (Yu et al., 2020), and LsNRL4 regulating photosynthesis and leaf angles (An et al., 2022). Unfortunately, these genes do not contain shared DMRs.*

*While the limited progress in lettuce domestication has hindered our further analysis of the methylation status of domestication traits-associated genes, we have identified hundreds of genes that are associated with these domestication-induced DMRs, which will serve as valuable resources to facilitate future functional genomic studies in investigating domestication genes in lettuce.*

*Relevant references*

*An G, Qi Y, Zhang W, Gao H, Qian J, Larkin RM, Chen J, Kuang H: LsNRL4 enhances photosynthesis and decreases leaf angles in lettuce. Plant Biotechnol. J. 2022, 20:1956-1967.*

*Luo C, Wang S, Ning K, Chen Z, Wang Y, Yang J, Qi M, Wang Q: The APETALA2 transcription factor LsAP2 regulates seed shape in lettuce. J. Exp. Bot. 2021, 72:2463-2476.*

*Wei T, van Treuren R, Liu X, Zhang Z, Chen J, Liu Y, et al: Whole-genome resequencing of 445 Lactuca accessions reveals the domestication history of 24 cultivated lettuce. Nat. Genet. 2021, 53:752-760.*

*Yu C, Yan C, Liu Y, Liu Y, Jia Y, Lavelle D, An G, Zhang W, Zhang L, Han R, et al: Upregulation of a KN1 homolog by transposon insertion promotes leafy head development in lettuce. Proc. Natl. Acad. Sci. USA 2020, 117:33668-33678.*

>Reviewer:

11. Line 279, "DNA methylation changes significantly induced expression changes of both DPGs and DDGs in stem lettuce". Whether the hyper-DMRs or hypo-DMRs induced gene expression? It is suggested that the author divide into two categories (hyper-DMRs or hypo-DMRs) to see.

*>Author:*

*We thank the reviewer for this suggestion. In the revised manuscript, we have further analyzed gene expression changes of DPGs and DDGs related to hyper- and hypo-DMRs (Additional file 2: new Fig. S10) and found that both hyper DMRs and hypo-DMRs significantly influence expression of DPGs and DDGs in stem lettuce.*

>Reviewer:

12. Line 282, why the authors selected Lsat_1_v5_gn_1_50480 and Lsat_1_v5_gn_1_50600 as case genes? What roles do Lsat_1_v5_gn_1_50480 and Lsat_1_v5_gn_1_50600 play during lettuce domestication? It should verify the expression regulation of DNA methylation for candidate genes using 5-Aza or LUC/REN, and the function of candidate genes.

*>Author:*

*Lsat_1_v5_gn_1_50480 and Lsat_1_v5_gn_1_50600 were shown as the former is an identified DPG, and the latter is a DDG this is associated with the same shared hypo-DMR, serving as examples to show that DNA methylation changes influence expression changes of both DPGs and DDGs. The biological functions of these two genes are not characterized in lettuce, while the Arabidopsis homolog of Lsat_1_v5_gn_1_50480 (AT5G04550) is possibly involved in abiotic stress responses (Luhua et al., 2013). We have involved this information in the revised sentence.*

*Furthermore, we performed 5-azacytidine (5-AzaC) treatment on the wild lettuce (L. serriola) seedlings to verify the influence of DNA methylation on the expression levels of these two genes, as suggested by this reviewer. We found that 5-AzaC treatment results in significantly decreased DNA methylation in the candidate region (Chr1: 59,076,400-59,077,800; Fig. 6c) in wild lettuce and elevated expression of both of Lsat_1_v5_gn_1_50480 and Lsat_1_v5_gn_1_50600 (Additional file 2: new Fig. S11), further supporting the effect of DNA methylation on the expression levels of DPGs and DDGs. The relevant Figure and descriptions have been included in the revised manuscript.*

*Relevant reference*

*Luhua S, Hegie A, Suzuki N, Shulaev E, Luo X, Cenariu D, Ma V, Kao S, Lim J, Gunay MB, et al: Linking genes of unknown function with abiotic stress responses by high-throughput phenotype screening. Physiol. Plant. 2013, 148:322-333.*

**Second round of review**

**Reviewer 1**

The authors did a good job to perform more experiments and analysis to address most of the questions. There are still some problems that concern me.

The first is lower read coverage. The author cited a reference to claim that the manuscript has enough coverage. However, the reads were used for different purpose in the two papers. The cited reference only used low-depth reads to analyze genome-wide methylation profiles, not using it to call DMRs, while this manuscript used low-depth reads for DMR calling. The authors should use other methods (e.g. PCR) to confirm the reliability of the DMRs.

The other is that the RNA-seq data is not matching the methylation data. It is known that DNA methylation and RNA expression varies across genotypes/accessions and tissues. Therefore, it is necessary to use RNAseq and methylation data from same tissues of same genotypes. The author should at least use qRT-PCR to confirm the conclusions in Figure 6.

Two minor questions:

1, Reply to original question 3, I suggest the authors to include this in the discussion (Whether the genes associated with shared hyper- or hypo- DMRs were under selection).

2, P5, it should be 24 accessions of wild relatives, not 34.

**Reviewer 2**

Manuscript of "Evolution of DNA methylation during lettuce domestication and divergence" analyzed the DNA methylation variation during lettuce domestication and divergence based on 52 lettuce accessions. This is the manuscript that I reviewed, but unfortunately, the author did not address all of my concerns. As a result, the existing results are insufficient to support the conclusion.

Major comments:

1. In line 109-111, the authors removed cytosines at single nucleotide polymorphism (SNP) loci. This approach may seem violent. However, most previous studies constructed "pseudo-reference" to eliminate the SNP's effect, such as in Arabidopsis thaliana (https://doi.org/10.1016/j.cell.2016.06.044), soybean (https://doi.org/10.1186/s13059-018-1516-z), tomato (10.1007/s11427-022-2299-5), maize (https://doi.org/10.1038/s41467-020-19333-4). The authors should carefully evaluate the suitability of these two approaches for the current study. Alternatively, they could conduct a comparative analysis to assess the variance in results obtained from employing each approach.

2. In line 270-280. The biological functions of these two genes (Lsat_1_v5_gn_1_50480 and Lsat_1_v5_gn_1_50600) are not characterized in lettuce. The authors did not verify the function of these two genes. The authors discussed that domestication-induced DNA methylation variations influence gene expression. However, the evidence related the roles of DNA methylation during lettuce domestication, particularly as related to trait changes, is lacked. It is necessary for the author to provide additional experimental evidence to substantiate their claims. If they are unable to do so, the current results are inadequate to support the conclusion.

Minor comments:

3. Authors found a significant increase of DNA methylation during lettuce domestication, but what effect does this increase have on trait during lettuce domestication? For example, some genes located nearby DMRs might associate with lettuce domesticated traits. The author can discuss this in the discussion.

4. What software did the author use to identify the DMR in this study?

5. What are the parameters for SNPs filtering? missing rate? MAF?

6. The authors should provide more detailed information in the methods part. For example, additional details about the analysis of RNA-seq would be beneficial.

7. The authors should provide the methods of Bisulfite-Seq PCR analysis

**Authors Response**

**Point-by-point responses to the reviewers’ comments:**

Reviewer #1

The authors did a good job to perform more experiments and analysis to address most of the questions. There are still some problems that concern me.

>Reviewer:

The first is lower read coverage. The author cited a reference to claim that the manuscript has enough coverage. However, the reads were used for different purpose in the two papers. The cited reference only used low-depth reads to analyze genome-wide methylation profiles, not using it to call DMRs, while this manuscript used low-depth reads for DMR calling. The authors should use other methods (e.g. PCR) to confirm the reliability of the DMRs.

*>Author:*

*As suggested, we have performed quantitative real-time PCR using MspJI-digested methylated genomic DNAs following the previous described approach (Eichten SR et al, 2011). We randomly chose 36 domestication-induced DMRs including 18 hyper- and 18 hypo-DMRs located on 9 chromosomes, and 18 hyper- and 16 hypo-DMRs were confirmed (Additional file 1: new Tables S4 and S15). These results confirm the reliability of the identified DMRs.*

*Relevant reference*

*Eichten SR, Swanson-Wagner RA, Schnable JC, Waters AJ, Hermanson PJ, Liu S, Yeh C-T, Jia Y, Gendler K, Freeling M, et al: Heritable epigenetic variation among maize inbreds. PloS Genetics 2011, 7:e1002372.*

>Reviewer:

The other is that the RNA-seq data is not matching the methylation data. It is known that DNA methylation and RNA expression varies across genotypes/accessions and tissues. Therefore, it is necessary to use RNAseq and methylation data from same tissues of same genotypes. The author should at least use qRT-PCR to confirm the conclusions in Figure 6.

*>Author:*

*We agree with this reviewer and have confirmed the changes of gene expression in cultivated lettuce compared to wild lettuce shown in Figure 6 using qRT-PCR analysis (Additional file 2: new Figure S12).*

Two minor questions:

>Reviewer:

1, Reply to original question 3, I suggest the authors to include this in the discussion (Whether the genes associated with shared hyper- or hypo- DMRs were under selection).

*>Author:*

*We thank the reviewer for this suggestion and have incorporated the relevant discussion in the Discussion section of the revised manuscript (Lines 380-389).*

>Reviewer:

2, P5, it should be 24 accessions of wild relatives, not 34.

*>Author:*

*We thank the reviewer for this correction and have revised it in the manuscript.*

Reviewer #2

Manuscript of "Evolution of DNA methylation during lettuce domestication and divergence" analyzed the DNA methylation variation during lettuce domestication and divergence based on 52 lettuce accessions. This is the manuscript that I reviewed, but unfortunately, the author did not address all of my concerns. As a result, the existing results are insufficient to support the conclusion.

Major comments:

>Reviewer:

1. In line 109-111, the authors removed cytosines at single nucleotide polymorphism (SNP) loci. This approach may seem violent. However, most previous studies constructed “pseudo-reference” to eliminate the SNP’s effect, such as in Arabidopsis thaliana, soybean, tomato (10.1007/s11427-022-2299-5), maize. The authors should carefully evaluate the suitability of these two approaches for the current study. Alternatively, they could conduct a comparative analysis to assess the variance in results obtained from employing each approach.

*>Author:*

*As suggested, we have used the approach described in previous studies (Cao et al, 2022; Guo et al., 2023) to mask the reference genome using SNPs to construct a “pseudo-reference” for mapping WGBS data from butterhead lettuce (CLBH_1-5) and wild accessions (WLLS_1-15) (Additional file 1: Table S1). The average uniquely mapping rates based on the pseudo-reference of butterhead and wild lettuce were 75.9% and 67.9%, respectively, which are comparable to 75.6% and 67.1% achieved using our current method. Similarly, the average global CG methylation levels based on pseudo-reference of butterhead and wild lettuce were 89.9% and 85.1%, respectively, which are also comparable to 89.8% and 85.1% based on our current method. Moreover, the DMRs identified in wild lettuce compared to butterhead based on the pseudo-reference were consistent with those identified by the current method. These observations suggest that compared to the pseudo-reference approach, our current approach is equally reliable in identifying DNA methylation variations while excluding the effects of SNPs.*

*Relevant references*

*Cao S, Wang L, Han T, Ye W, Liu Y, Sun Y, et al: Small RNAs mediate transgenerational inheritance of genome-wide trans-acting epialleles in maize. Genome Biol. 2022, 23:53.*

*Guo H, Cao P, Wang C, Lai J, Deng Y, Li C, Hao Y, Wu Z, Chen R, Qiang Q, et al: Population analysis reveals the roles of DNA methylation in tomato domestication and metabolic diversity. Sci. China Life Sci. 2023, 66:1888-1902.*

>Reviewer:

2. In line 270-280. The biological functions of these two genes (Lsat_1_v5_gn_1_50480 and Lsat_1_v5_gn_1_50600) are not characterized in lettuce. The authors did not verify the function of these two genes. The authors discussed that domestication-induced DNA methylation variations influence gene expression. However, the evidence related the roles of DNA methylation during lettuce domestication, particularly as related to trait changes, is lacked. It is necessary for the author to provide additional experimental evidence to substantiate their claims. If they are unable to do so, the current results are inadequate to support the conclusion.

*>Author:*

*We thank the reviewer for this suggestion.*

*First, we would like to clarify that the two genes, Lsat_1_v5_gn_1_50480 and Lsat_1_v5_gn_1_50600, were chosen as examples to show the influence of domestication-induced DNA methylation variations on the expression of both DMR-associated proximal genes (DPGs) and DMR-associated distal genes (DDGs). This influence has also been verified using 5-AzaC treatment following the previous suggestion of the reviewer (Additional file 2: Figure S13). We are not attempting to correlate changes in the expression of these two genes with lettuce domestication traits. To avoid confusion, we have modified our conclusion of this subsection as “These results indicate that variations in DNA methylation may affect chromatin accessibility and hence alter expression levels of the associated proximal and distal genes.”, and revised the relevant sentence in the Abstract in the revised manuscript. In addition, we did some preliminary examination of the function of Lsat_1_v5_gn_1_50600 (homolog of AT5G04550 annotated to be probably related to abiotic stress responses) in N. benthamiana, and found that it had no obvious effect in response to salt stress. Its functional verification on lettuce developmental and other traits may require the generation of loss-of-function mutants in lettuce, which may take an additional 1-2 years.*

*Second, to address the reviewer’s concern regarding the lack of evidence relating the roles of DNA methylation variations in trait changes during lettuce domestication, we have further examined the function of LsERF48, a transcription factor we identified to be related with domestication-induced DMRs (Figure 5; Additional file 2: Figure S8). 35S:LsERF48-GFP, but not the 35S:GFP control, reduced ROS accumulation in response to salt stress in N. benthamiana (Additional file 2: new Figure S9), suggesting the role of LsERF48 in enhancing salt stress tolerance. It has been suggested that the domestication process is driven significantly by the adaptation of stress tolerance to human-provided environments and conditions, leading to considerable changes in morphology, behavior, and physiology (Chen et al., 2015; Razzaq et al., 2021). Notably, cultivated crops grown in human-manipulated environments are often accompanied by tradeoffs in stress resistance to promote growth and yield (Li et al., 2017; Wang et al., 2020). Thus, we believe that the involvement of the identified epigenetic variations in stress responses is worth further exploration.*

*Third, we have added the relevant discussion on the possible involvement of DNA methylation variations in trait changes in lettuce domestication in the “Discussion” section of the revised manuscript (Lines 380-403). With the above clarifications and new results, we hope the reviewer agree that the thousands of genes associated with DNA methylation variations in lettuce domestication and divergence will serve as valuable resources to facilitate future functional genomic studies to investigate traits-associated genes in lettuce evolution.*

*Relevant references*

*Chen, Y.H., Gols, R., and Benrey, B. Crop domestication and its impact on naturally selected trophic interactions. Annu. Rev. Entomol. 2015, 60: 35-58.*

*Li LF, Li YL, Jia Y, Caicedo AL, Olsen KM: Signatures of adaptation in the weedy rice genome. Nat. Genet. 2017, 49:811-814.*

*Razzaq, A., Saleem, F., Wani, S.H., Abdelmohsen, S.A.M., Alyousef, H.A., Abdelbacki, A.M.M., Alkallas, F.H., Tamam, N., and Elansary, H.O. De-novo Domestication for Improving Salt Tolerance in Crops. Front. Plant Sci. 2021, 12: 681367.*

*Wang L, Cao S, Wang P, Lu K, Song Q, Zhao FJ, et al: DNA hypomethylation in tetraploid rice potentiates stress-responsive gene expression for salt tolerance. Proc. Natl. Acad. Sci. USA 2021, 118.*

Minor comments:

>Reviewer:

3. Authors found a significant increase of DNA methylation during lettuce domestication, but what effect does this increase have on trait during lettuce domestication? For example, some genes located nearby DMRs might associate with lettuce domesticated traits. The author can discuss this in the discussion.

*>Author:*

*We thank this reviewer for this suggestion and have added the relevant discussion in the “Discussion” section of the revised manuscript (Lines 380-403).*

>Reviewer:

4. What software did the author use to identify the DMR in this study?

*>Author:*

*DMRs were identified using DMRfinder (v0.3) (Gaspar et al, 2017), in conjunction with R language (V4.3.1) for statistical tests and adjusting p-values. This information has been included in the revised manuscript.*

*Relevant reference*

*Gaspar JM, Hart RP. DMRfinder: efficiently identifying differentially methylated regions from MethylC-seq data. BMC Bioinformatics. 2017, 18:528.*

>Reviewer:

5. What are the parameters for SNPs filtering? missing rate? MAF?

*>Author:*

*The parameters of > 40% missing calls and minor allele frequency (MAF) < 0.05 were used to filter the SNPs. We have included this information in the revised manuscript.*

>Reviewer:

6. The authors should provide more detailed information in the methods part. For example, additional details about the analysis of RNA-seq would be beneficial.

*>Author:*

*We thank the reviewer for this suggestion and included more detailed information for RNA-seq and Hi-C data analysis in the revised manuscript.*

>Reviewer:

7. The authors should provide the methods of Bisulfite-Seq PCR analysis.

*>Author:*

*As suggested, we have provided the detailed method for analyzing DNA methylation levels by qPCR under the subsection of “Analysis of DMRs using qPCR” in the Method section of the revised manuscript.*

**Third round of review**

**Reviewer 1**

The authors have answered all my questions. I don't have any other questions.

**Reviewer 2**

Manuscript of "Evolution of DNA methylation during lettuce domestication and

divergence" analyzed the DNA methylation variation during lettuce domestication and

divergence based on 52 lettuce accessions. This is the manuscript previously reviewed and resubmitted. However, there are still some problems should be concerned.

1. Most previous studies related to population methylation constructed "pseudo-reference" to eliminate the SNP's effect, such as publications about Arabidopsis thaliana (http://dx.doi.org/10.1016/j.cell.2016.06.044), soybean (https://doi.org/10.1186/s13059-018-1516-z), tomato (10.1007/s11427-022-2299-5), and maize (10.1038/s41467-020-19333-4). However, the authors selected only two lettuces to investigate the role of the "pseudo-reference", which is inherently random. It is better to use "pseudo-reference" base on all of accessions, and to see if there is a significant improvement from the author's method.

2. For our second question in Second Round of review, the authors provided the results of transient expression experiments in N. benthamiana leaves. The experimental evidence is weak. The authors said that they are not attempting to correlate changes in the expression of genes with lettuce domestication traits, but they try to reveal the influence of DNA methylation on gene expression. Furthermore, RNA-seq data is not matching the methylation data. The RNA-seq data were download from previous study, and 2.5-month-old plants were used to RNA-seq in previous study (https://doi.org/10.1038/s41467-017-02445-9). The WGBS data were obtained in this study, and 30 days after planting were collected for WGBS. In additional, the tissues sampled in the two studies were also not identical (Previous study: the top fully expanded leaves; this study: the third pair of leaves). As we known, DNA methylation and RNA-seq exhibited higher tissues specificity. Therefore, we are afraid that the conclusion reported in this manuscript could be affected by different samples used for data collecting.

**Authors Response**

**Point-by-point responses to the reviewers’ comments:**

Reviewer #1

The authors have answered all my questions. I don't have any other questions.

*>Author:*

*We appreciate the supportive comments from this reviewer and previous suggestions for improving this manuscript.*

Reviewer #2

Manuscript of "Evolution of DNA methylation during lettuce domestication and divergence" analyzed the DNA methylation variation during lettuce domestication and divergence based on 52 lettuce accessions. This is the manuscript previously reviewed and resubmitted. However, there are still some problems should be concerned.

*>Author:*

*We appreciate the insightful suggestions from the reviewer for improving the manuscript.*

>Reviewer:

1. Most previous studies related to population methylation constructed "pseudo-reference" to eliminate the SNP's effect, such as publications about Arabidopsis thaliana, soybean, tomato (10.1007/s11427-022-2299-5), and maize (10.1038/s41467-020-19333-4). However, the authors selected only two lettuces to investigate the role of the "pseudo-reference", which is inherently random. It is better to use "pseudo-reference" base on all of accessions, and to see if there is a significant improvement from the author's method.

*>Author:*

*We thank the reviewer for this suggestion. As suggested, we have used the “pseudo-reference” approach (Xu et al, 2020; Cao et al, 2022; Guo et al., 2023) to map our WGBS data of all accessions. The average uniquely mapping rates based on the pseudo-reference were 77.73% (crisp), 75.91% (butterhead), 75.96% (cos), 75.68% (stem), and 67.96% (wild lettuce), which is slightly higher to those achieved using the method of removing SNPs at cytosines: 77.73% (crisp), 75.64% (butterhead), 75.94% (cos), 75.63% (stem), and 67.19% (wild lettuce). The average global CG methylation levels based on pseudo-reference were 90.43% (crisp), 89.85% (butterhead), 90.64% (cos), 89.21% (stem), and 85.13% (wild lettuce), which were comparable to those based on the method of removing SNPs at cytosines: 90.43% (crisp), 89.84% (butterhead), 90.64% (cos), 89.21% (stem), 85.12% (wild lettuce). The DMRs identified in wild lettuce compared to cultivated lettuce based on the pseudo-reference were consistent with those identified by the method of removing SNPs at cytosines.*

*Nevertheless, compared to the approach of removing SNPs at cytosines used in our previous manuscript, utilizing the pseudo-reference approach slightly improves mapping rates and facilitates the acquisition of more effective reads for subsequent analysis. Therefore, in the revised manuscript, we employed the pseudo-reference approach to reanalyze all our WGBS data and updated all main Figures and relevant Supplemental Figures (S1-S7) and Supplemental Tables (S1, S2, S3, S14). As nearly all DMRs identified using the pseudo-reference remained the same as those identified using our previous approach, our conclusions remained consistent.*

*In the revised manuscript, we have also revised the relevant texts (Lines 105-109) as follows: “To exclude the effect of genomic variations on the DNA methylation analysis, we used single nucleotide polymorphisms (SNPs) of each accession to construct their corresponding pseudo-reference genome for subsequent methylation analysis [30, 35]. Nearly 600 Gb of WGBS data of all accessions were mapped to pseudo-reference genomes.”. In addition, we have also revised the methods of “Single cytosine methylation calling and population parameter (Pi, π)” in the “Methods” part (Lines 473-476).*

*Relevant references*

*Xu G, Lyu J, Li Q, Liu H, Wang D, Zhang M, Springer NM, Ross-Ibarra J, Yang J: Evolutionary and functional genomics of DNA methylation in maize domestication and improvement. Nat. Commun. 2020, 11:5539.*

*Cao S, Wang L, Han T, Ye W, Liu Y, Sun Y, et al: Small RNAs mediate transgenerational inheritance of genome-wide trans-acting epialleles in maize. Genome Biol. 2022, 23:53.*

*Guo H, Cao P, Wang C, Lai J, Deng Y, Li C, Hao Y, Wu Z, Chen R, Qiang Q, et al: Population analysis reveals the roles of DNA methylation in tomato domestication and metabolic diversity. Sci. China Life Sci. 2023, 66:1888-1902.*

>Reviewer:

2. For our second question in Second Round of review, the authors provided the results of transient expression experiments in N. benthamiana leaves. The experimental evidence is weak. The authors said that they are not attempting to correlate changes in the expression of genes with lettuce domestication traits, but they try to reveal the influence of DNA methylation on gene expression. Furthermore, RNA-seq data is not matching the methylation data. The RNA-seq data were download from previous study, and 2.5-month-old plants were used to RNA-seq in previous study. The WGBS data were obtained in this study, and 30 days after planting were collected for WGBS. In additional, the tissues sampled in the two studies were also not identical (Previous study: the top fully expanded leaves; this study: the third pair of leaves). As we known, DNA methylation and RNA-seq exhibited higher tissues specificity. Therefore, we are afraid that the conclusion reported in this manuscript could be affected by different samples used for data collecting.

*>Author:*

*We thank the reviewer for this suggestion. As suggested, we have performed RNA-seq of all accessions of wild and cultivated lettuce used in our study (Additional file 1: new Table S2). RNA was extracted from the same batch of plant tissues (the third pairs of leaves at 30 days after planting) used for DNA extraction. Using our own RNA-seq data, we observed consistent trends of gene expression changes (new Figure 6b,c; Additional file 2: Figure S6) as described in our previous manuscript. These new results further demonstrate the influence of DNA methylation on gene expression. Moreover, all RNA-seq data generated in this study were also deposited into the Genome Sequence Archive in BIG Data Center under the accession number PRJCA017183.*

*In the revised manuscript, the detailed method for RNA-seq library construction and analysis has been included in the “Methods” part as follows: “Total RNA was extracted from the same batch of plant tissues used for MethylC-seq and DNA-seq analysis. RNA-seq libraries were constructed as previously reported [82]. After DNase I treatment, aliquots of total RNA were individually fragmented. First-strand cDNA synthesis was performed using oligo(dT)-index primer, followed by second strand cDNA synthesis, end-repair, and adapter ligation. Subsequently, cDNA was amplified by 15 cycles of PCR reaction using Q5 HiFi HotStart DNA Polymerase (NEB). After purification, the 3’ RNA-seq libraries were sequenced on NovaSeq platform (Illumina) to generate 150 bp paired-end reads. After filtering with fastp (v0.23.2) [72], clean reads were mapped to the lettuce reference genome (Salinas_v8) using Hisat2 (v2.2.1) [85]. After removing potential PCR duplicates using Picard-tools (version 2.0.1), uniquely mapped reads on each gene were normalized as Reads per Million mapped reads (RPM) to represent the expression level for each gene using featureCounts (v2.0.4) [86] with default parameters.”*

**Fourth round of review**

**Reviewer 2**

The authors have answered all my questions. I don't have any other questions.
